# A novel protein RASON encoded by a lncRNA controls oncogenic RAS signaling in KRAS mutant cancers

**DOI:** 10.1038/s41422-022-00726-7

**Published:** 2022-10-14

**Authors:** Rongjie Cheng, Fanying Li, Maolei Zhang, Xin Xia, Jianzhuang Wu, Xinya Gao, Huangkai Zhou, Zhi Zhang, Nunu Huang, Xuesong Yang, Yaliang Zhang, Shunli Shen, Tiebang Kang, Zexian Liu, Feizhe Xiao, Hongwei Yao, Jianbo Xu, Chao Yan, Nu Zhang

**Affiliations:** 1 State Key Laboratory of Pharmaceutical Biotechnology, School of life Sciences, Nanjing University, Nanjing, Jiangsu China; 2 Department of Neurosurgery, The First Affiliated Hospital of Sun Yat-sen University, Guangzhou, Guangdong China; 3 Institute of Molecular Enzymology, School of Biology and Basic Medical Sciences, Soochow University, Suzhou, Jiangsu China; 4 Department of Hepatological surgery, The First Affiliated Hospital of Sun Yat-sen University, Guangzhou, Guangdong China; 5 State Key Laboratory of Oncology in South China, Collaborative Innovation Center for Cancer Medicine, Sun Yat-sen University Cancer Center, Guangzhou, Guangdong China; 6 Department of Scientific Research Section, The First Affiliated Hospital of Sun Yat-sen University, Guangzhou, Guangdong China; 7 Department of Gastrointestinal surgery, The First Affiliated Hospital of Sun Yat-sen University, Guangzhou, Guangdong China; 8 Chemistry and Biomedicine Innovation Center, Institute of Artificial Intelligence Biomedicine, Nanjing University, Nanjing, Jiangsu China; 9 Engineering Research Center of Protein and Peptide Medicine, Ministry of Education, Nanjing, Jiangsu China; 10 Institute of Pancreatology, Nanjing University, Nanjing, China; 11 Guangdong Provincial Key Laboratory of Brain Function and Disease, Guangzhou, Guangdong China

**Keywords:** Gastrointestinal cancer, Cancer therapy

## Abstract

Mutations of the *RAS* oncogene are found in around 30% of all human cancers yet direct targeting of RAS is still considered clinically impractical except for the KRAS^G12C^ mutant. Here we report that RAS-ON (RASON), a novel protein encoded by the long intergenic non-protein coding RNA 00673 (*LINC00673*), is a positive regulator of oncogenic RAS signaling. RASON is aberrantly overexpressed in pancreatic ductal adenocarcinoma (PDAC) patients, and it promotes proliferation of human PDAC cell lines in vitro and tumor growth in vivo. CRISPR/Cas9-mediated knockout of *Rason* in mouse embryonic fibroblasts inhibits KRAS-mediated tumor transformation. Genetic deletion of *Rason* abolishes oncogenic KRAS-driven pancreatic and lung cancer tumorigenesis in *LSL-Kras*^*G12D*^*; Trp53*^*R172H/+*^ mice. Mechanistically, RASON directly binds to KRAS^G12D/V^ and inhibits both intrinsic and GTPase activating protein (GAP)-mediated GTP hydrolysis, thus sustaining KRAS^G12D/V^ in the GTP-bound hyperactive state. Therapeutically, deprivation of RASON sensitizes KRAS mutant pancreatic cancer cells and patient-derived organoids to EGFR inhibitors. Our findings identify RASON as a critical regulator of oncogenic KRAS signaling and a promising therapeutic target for KRAS mutant cancers.

## Introduction

The human *RAS* gene family includes *KRAS*, *HRAS* and *NRAS*, all of which encode 21 kDa small GTPase proteins that are activated on the inner cell membrane and transduce extracellular growth stimuli signals to intracellular effector signaling cascades.^1^ RAS proteins are binary molecular switches that cycle between an ‘ON’ state (GTP bound) and an ‘OFF’ state (GDP bound). The subtle balance between these two states is mainly controlled by guanine nucleotide exchange factors (GEFs), which convert RAS-GDP to RAS-GTP; and GTPase-activating proteins (GAPs), which convert RAS-GTP back to RAS-GDP through hydrolysis.^2^

Activating *RAS* mutations are found in around 30% of all human cancers.^3,4^ Among the three *RAS* genes, mutation frequency of *KRAS* in human cancer is considerably higher than that of *HRAS* and *NRAS*. For example, *KRAS*^*G12C*^ mutation is detected in around 12%–15% of non-small cell lung cancer (NSCLC) patients; whereas more than 90% of pancreatic ductal adenocarcinoma (PDAC) patients bear *KRAS* mutations, especially the *KRAS*^*G12D*^ and *KRAS*^*G12V*^ subtypes.^5^ These mutations are thought to impair both the intrinsic GTPase activity of KRAS-GTP and the rate of GAP-mediated GTP hydrolysis in KRAS-GTP,^6^ thus leading to constitutively active oncogenic KRAS signaling.

Despite the critical role of RAS signaling in human cancers, direct targeting of the RAS protein has remained a great challenge for the past forty years. Alternative therapies against the upstream growth factor receptors such as EGFR have been applied in NSCLC and colon cancer, but those patients with RAS mutations were excluded.^7^ Moreover, secondary resistance to EGFR inhibitors in patients with wild-type RAS is often driven by acquired RAS mutations.^8^ On the other hand, targeting RAS downstream effectors, including RAF and MEK, showed limited benefits in RAS-mutant cancers.^9^ Very recently, small-molecular compounds that could directly target KRAS^G12C^ mutant through covalent binding have been approved for the treatment of NSCLC.^10–14^ However, strategies for targeting other KRAS mutations such as KRAS^G12D^ and KRAS^G12V^, which are the prevalent mutation subtypes in PDAC, are desperately needed. Here we report the discovery of a novel protein, termed RAS-ON (RASON), that plays a critical role in oncogenic RAS signaling and represents a vulnerable therapeutic target for mutant KRAS-driven cancers.

## Results

### LINC00673 encodes a novel protein RASON

**Fig. 1:**
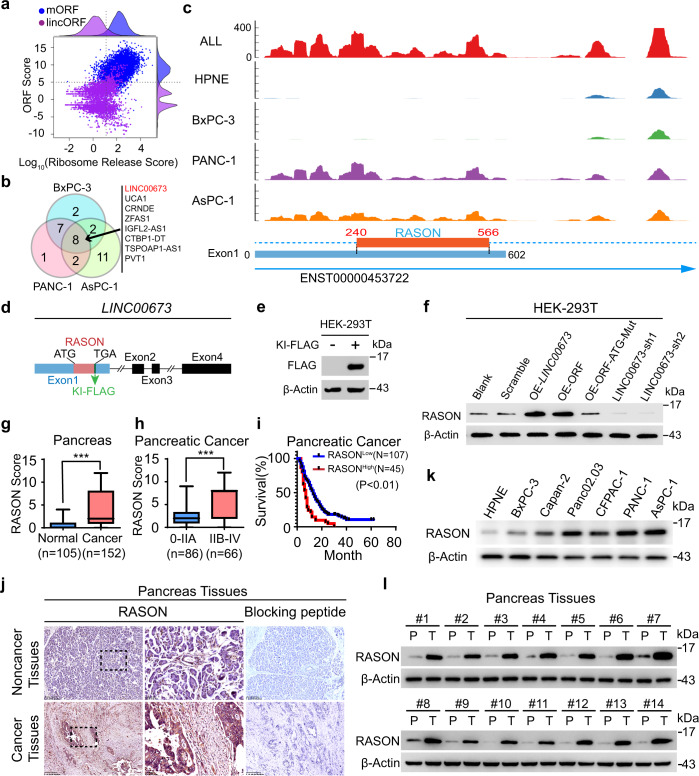
RASON is overexpressed in human pancreatic cancer. **a** Scatter plot showing the ribosome release score (RSS) and the ORF score from main ORF (mORF, blue) and lincORF (purple) (*x*-axis, Log_10_ RSS; *y*-axis, ORF score). The dashed lines represent the 95% percentile of RSS and ORF scores from mORF, which are set as the threshold to identify actively translated lincORF. Along each axis, all points are summarized using an overlaid density plot. **b** Venn diagram of differentially translated lincRNAs in three PDAC cell lines BxPC-3, PANC-1, and AsPC-1 compared to HPNE normal cells by Ribo-seq. **c** Upper, differential RPFs of RASON sORF between HPNE and PDAC cells; lower, diagram of the sORF encoding RASON. **d** Scheme of CRISPR/Cas9-induced FLAG-tag insertion after RASON sORF inside *LINC00673* genomic DNA in 293T cells. **e** IB showing successful expression of RASON fused with the FLAG tag in **d**. **f** RASON protein expression in 293T cells transfected with LINC00673, RASON-ORF, RASON-ORF with ATG mutation, or sh-LINC00673. **g**, **h** Correlation analysis of RASON expression with tumor grade in 152 PDAC patients. Data are means ± SD. **i** Kaplan-Meier curve showing overall survival of patients stratified by RASON expression in the 152 PDAC patient cohort. **j** Representative IHC images of RASON expression in tumors from 105 PDAC patients, with blocking peptide showing the specificity of anti-RASON antibody (bars, 250 µm or 50 µm). **k** IB of RASON expression in a panel of PDAC cell lines. **l** RASON protein expression levels in paired cancerous and normal tissues (>2 cm away) from 14 randomly selected PDAC patients. Data in bar graphs are shown as means ± SD. *P* values were calculated by Wilcoxon test (**g**, **h**) and log-rank analysis (**i**). ***P < 0.001.

Advances in genome-wide translatome lead to the identification of hundreds of functional peptides or proteins from noncoding regions of the genome, including long intergenic noncoding RNAs (lincRNAs), 5′ un-translational region (5′-UTR) and pri-micro RNAs.^15–17^ We screened small open reading frame (sORF) in lincRNAs that could encode novel proteins in PDAC by performing ribosome-sequencing in the normal pancreatic ductal cell line HPNE and three PDAC cell lines BxPC-3, PANC-1 and AsPC-1. Certain Ribosome Protected Fragments (RPFs) were mapped to transcripts annotated as non-coding regions including 5′-UTR, 3′-UTR (of known protein-coding genes) and lincRNAs (Supplementary information, Fig. S1a). A total of 14,504 sORFs were identified with read counts ≥1, with an average length of 100 bp (Supplementary information, Fig. S1b). To identify active translation, the ribosome release score (RRS) and modified ORF score for each lincORF were calculated (Fig. 1a). Forty actively translated lincORFs were retained and subjected to differential analysis. Compared with HPNE cells, eight lincRNAs were found differentially translated in all three PDAC cell lines and LINC00673 ranked top of these candidates (Fig. 1b). Inspection of the LINC00673 transcript revealed an sORF in exon 1 that may encode a 108 amino acid protein (referred to as RASON hereafter), with differential RPFs between HPNE and PDAC cells (Fig. 1c). We also searched several published ribosome-sequencing datasets, RPFs of RASON were detected as well (Supplementary information, Fig. S1c). Using cohorts from TCGA and GTEx databases, we found that LINC00673 RNA was highly expressed in PDAC and several other cancer types (Supplementary information, Fig. S1d). We also enrolled twenty-four paired samples (tumor (T), adjacent normal tissue (N), and metastatic lymph node (M)) from eight PDAC patients and performed RNA-seq. A total of 834 differentially expressed lincRNAs were identified between N vs T, 959 between N vs M, and 13 between T vs M (Supplementary information, Fig. S1e). Among the eight differentially translated lincRNAs previously identified in PDAC cell lines (Fig. 1b), five were differentially expressed in N vs T and N vs M; LINC00673 also ranked top in these differentially expressed lincRNAs (Supplementary information, Fig. S1f).

To confirm the translation of RASON in cells, we generated a CRISPR/Cas9-induced FLAG tag insertion inside *LINC00673* genomic DNA in HEK293T cells, right after the *RASON* sORF (Fig. 1d; Supplementary information, Fig. S2a). Immunoblotting (IB) with anti-FLAG antibody confirmed the translation of a ~14 KD protein (Fig. 1e). RASON is highly conserved among primates, whereas mouse RASON (78 aa) has a shorter C-terminus but shares a fair homology with human RASON on the N-terminus (Supplementary information, Fig. S2b), we thus generated a rabbit monoclonal antibody against RASON (1–14 aa, Supplementary information, Fig. S2c) and performed IB in 293T cells overexpressing LINC00673, RASON-ORF, RASON-ORF with mutated ATG start codon, or shRNA against LINC00673 (sh-LINC00673). RASON expression was elevated after transfection of either LINC00673 or RASON-ORF but not RASON-ORF-ATG mutant and was inhibited by sh-LINC00673 (Fig. 1f). RASON-specific peptide sequences were also identified by mass spectrometry (MS) analysis in LINC00673-transfected 293T cells (Supplementary information, Fig. S2d), and endogenously in the AsPC-1 PDAC cell line (Supplementary information, Fig. S2e). Collectively, these data demonstrated the translation of RASON protein in human cells.

### RASON is aberrantly overexpressed in PDAC and correlates with poor prognosis

LINC00673 was identified as an oncogenic lncRNA in multiple human malignancies including PDAC.^18–22^ In an independent PDAC patient cohort including 105 PDAC cancerous samples and paired adjacent normal pancreatic samples, we confirmed that LINC00673 is highly expressed in cancerous tissues and its expression positively correlates with tumor grade (Supplementary information, Fig. S2f, g). Consistent with this, RASON protein expression is also significantly higher in cancerous tissues (Fig. 1g, the same patient cohort with 47 additional PDAC tumor samples) and positively correlates with both tumor grade (Fig. 1h) and poor overall survival (Fig. 1i). RASON expression levels were determined by immunohistochemistry (IHC) staining using the anti-RASON antibody, the specificity of which was confirmed by the addition of a blocking peptide (Fig. 1j). In PDAC cell lines, RASON expression is high in AsPC-1 (KRAS^G12D^) and PANC-1 (KRAS^G12D^) cells, and relatively low in BxPC-3 (KRAS^WT^) and Capan-2 (KRAS^G12V^) cells (Fig. 1k). The aberrant overexpression of RASON in human PDAC was also confirmed by IB in paired cancerous/adjacent normal tissue (>2 cm away) from 14 randomly selected PDAC patients (Fig. 1l). Taken together, these results suggested that RASON is an oncogenic protein and may serve as a prognostic marker for PDAC.

### RASON is involved in the regulation of oncogenic KRAS signaling

**Fig. 2:**
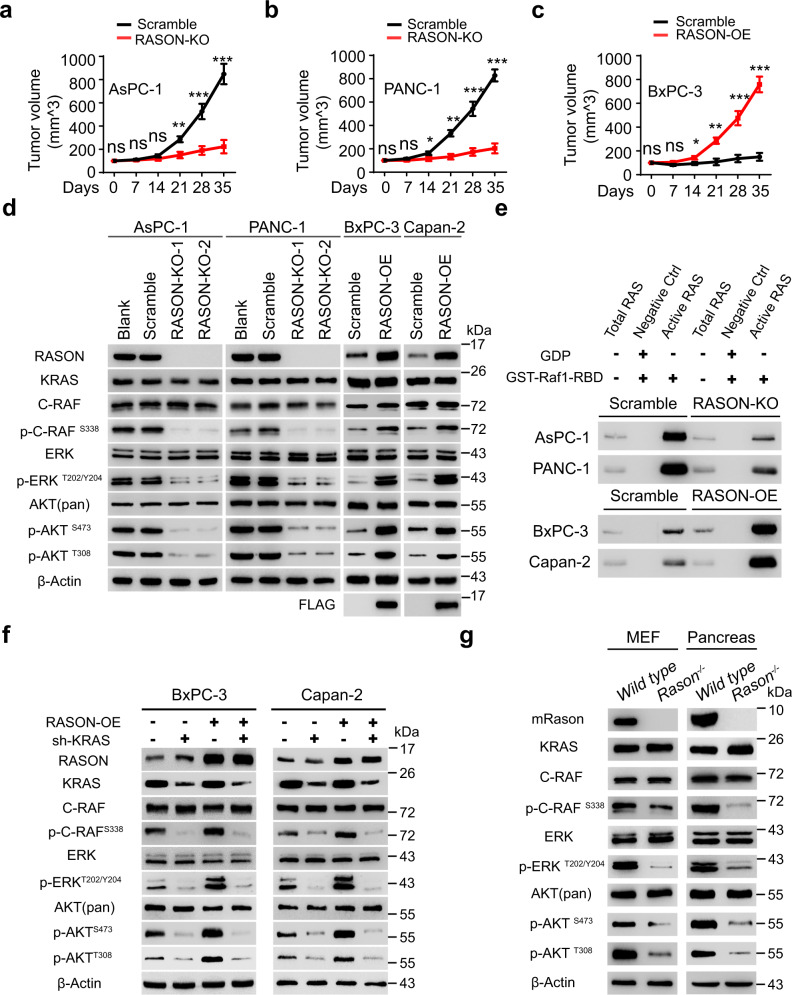
RASON is involved in the regulation of oncogenic KRAS signaling. **a**–**c** Xenograft tumor growth curves of RASON-KO AsPC-1 cells (**a**) and PANC-1 cells (**b**), and RASON-OE BxPC-3 cells (**c**). Data are means ± SEM. **d** Effects of RASON KO or OE on KRAS signaling pathway in PDAC cell lines. **e** Active KRAS-GTP levels as determined by GST-Raf1-RBD pull-down assay in two RASON-KO cells (AsPC-1 and PANC-1) and two RASON-OE cells (BxPC-3 and Capan-2). **f** IB showing the effects of KRAS KD and RASON OE on KRAS effector signaling in BxPC-3 and Capan-2 cells. **g** IB showing the status of KRAS effector signaling in MEFs or mouse pancreas from wild-type and *Rason*^−/−^ mice. *P* values were calculated by multiple *t*-test (**a**–**c**). *P < 0.05, **P < 0.01, ***P < 0.001.

We next investigated the potential biological function of RASON in PDAC. To differentiate the function of RASON protein from that of LINC00673 lincRNA, we used CRISPR/Cas9 to knock out only the *RASON* sORF region in AsPC-1 and PANC-1 cells (Supplementary information, Fig. S3a–c), which had neglectable effects on LINC00673 expression. On the other hand, BxPC-3 and Capan-2 cells with low RASON expression were stably overexpressed with LINC00673 or RASON-ORF (Supplementary information, Fig. S3d, e). Both AsPC-1 and PANC-1 RASON-knockout (KO) cells showed reduced cell proliferation in vitro and marked inhibition of xenograft tumor growth (Fig. 2a, b; Supplementary information, Fig. S4). In contrast, LINC00673 or RASON-ORF overexpression (OE) in BxPC-3 and Capan-2 cells enhanced tumor growth both in vitro and in vivo (Fig. 2c; Supplementary information, Fig. S5). Moreover, OE of RASON-ORF in LINC00673-knockdown (KD) AsPC-1 cells rescued most of the malignant phenotypes (Supplementary information, Fig. S6a–c), suggesting that RASON but not LINC00673 is the major executor of the protumor biological functions.

To elucidate the molecular mechanisms underlying the protumor function of RASON, we performed RNA-seq in AsPC-1 and PANC-1 RASON-KO cells. Both DEG and KEGG analysis implied the involvement of RAS downstream signaling including the MAPK (RAF-MEK-ERK) and PI3K-AKT pathways (Supplementary information, Fig. S6d). Immunoblotting experiments showed that both pathways were inhibited by RASON KO in AsPC-1 and PANC-1 cells, but enhanced by RASON OE in BxPC-3 and Capan-2 cells (Fig. 2d). While total KRAS level was not affected (Fig. 2d), active KRAS-GTP level was downregulated in AsPC-1 and PANC-1 cells by RASON KO, and upregulated in BxPC-3 and Capan-2 cells by RASON OE (Fig. 2e), as measured by a pull-down assay using the RAS binding domain of the RAS effector protein RAF1 (GST-Raf1-RBD). Moreover, KRAS KD by shRNA in BxPC-3 and Capan-2 cells abolished RASON OE-induced activation of MAPK and PI3K-AKT pathways, further demonstrating that RASON exerts its biological function through RAS signaling (Fig. 2f). Notably, the downregulation of RAS signaling in AsPC-1 cells by RASON KO could be readily rescued by the overexpression of mouse Rason (Supplementary information, Fig. S6e), suggesting an evolutionarily conserved mechanism.

RAS signaling is critical for the proliferation and migration of normal cells.^23^ To investigate whether RASON modulates RAS signaling in vivo, we generated *Rason* homologues KO mice by deletion of the entire sORF (Supplementary information, Fig. S7a). *Rason*^−/−^ mice are viable; however, mouse embryonic fibroblasts (MEFs) generated from prenatal *Rason*^−/−^ mice and pancreas tissues isolated from adult *Rason*^−/−^ mice both exhibited significant downregulation of RAS signaling, as evidenced by reduced phosphorylation of RAF and AKT, as well as reduced level of KRAS-GTP (Fig. 2g; Supplementary information, Fig. S7b, c). Together, the above results suggested that RASON is critical for tumor maintenance through activating RAS signaling.

### RASON is essential for KRAS-driven tumorigenesis

**Fig. 3:**
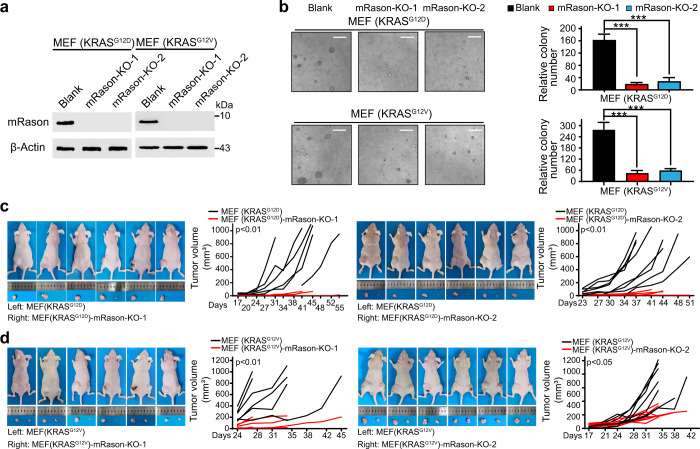
RASON is required for KRAS-mediated malignant transformation of MEF cells. **a** Mouse RASON protein expression level in RAS-less MEF cells stably transfected with KRAS^G12D^ or KRAS^G12V^ with or without *Rason* KO by CRISPR/Cas9. **b** Effect of *Rason* KO on the 3D anchorage-independent growth of KRAS^G12D^ or KRAS^G12V^ MEF cells. Representative images of colony formation were also shown (bars, 200 µm). **c**, **d** Effect of *Rason* KO on in vivo tumor formation (*n* = 6 mice) of KRAS^G12D^ (**c**) or KRAS^G12V^ (**d**) MEF cells. Two stable *Rason* KO clones were generated by CRISPR/Cas9 for each KRAS mutant. Representative images of mice with subcutaneous tumor were also shown. Data shown are means ± SD. *P* values were calculated by one-way ANOVA test (**b**). ***P < 0.001.

To explore whether RASON is also involved in KRAS-driven tumorigenesis, we used the RAS-less MEF cell lines^24^ stably transfected with KRAS^G12D^ or KRAS^G12V^ and tested the effect of *Rason* KO on KRAS-induced transformation of MEF cells. We generated two *Rason* KO clones for both KRAS^G12D^ and KRAS^G12V^ MEF cell lines using CRISPR/Cas9 (Fig. 3a). *Rason* KO almost completely diminished the tumorigenesis potential of KRAS^G12D^ or KRAS^G12V^ MEF cells in 3D anchorage-independent growth assays (Fig. 3b). In vivo subcutaneous tumor formation assays in nude mice also validated the critical role of RASON in KRAS^G12D^ or KRAS^G12V^-induced transformation of MEF cells, as *Rason* KO almost completely abolished the tumor formation compared with control cells implanted on the opposite side of the same mice (Fig. 3c, d). As expected, the suppression of RAS signaling by *Rason* KO in MEF cells could also be rescued by overexpression of human RASON (Supplementary information, Fig. S8a). To further validate RASON function in primary MEF cells, we isolated MEF cells from prenatal *LSL-Kras*^*G12D*^ mice and used a lentivirus construct to simultaneously activate *Kras*^*G12D*^ mutation (with Cre) and knock out *Rason* (with two sgRNAs, Supplementary information, Fig. S8b, c). *Rason* KO in these primary MEFs also suppressed malignant transformation induced by *Kras*^*G12D*^ mutation (Supplementary information, Fig. S8d), indicating that RASON is essential for KRAS-induced tumorigenesis in MEF cells.

**Fig. 4:**
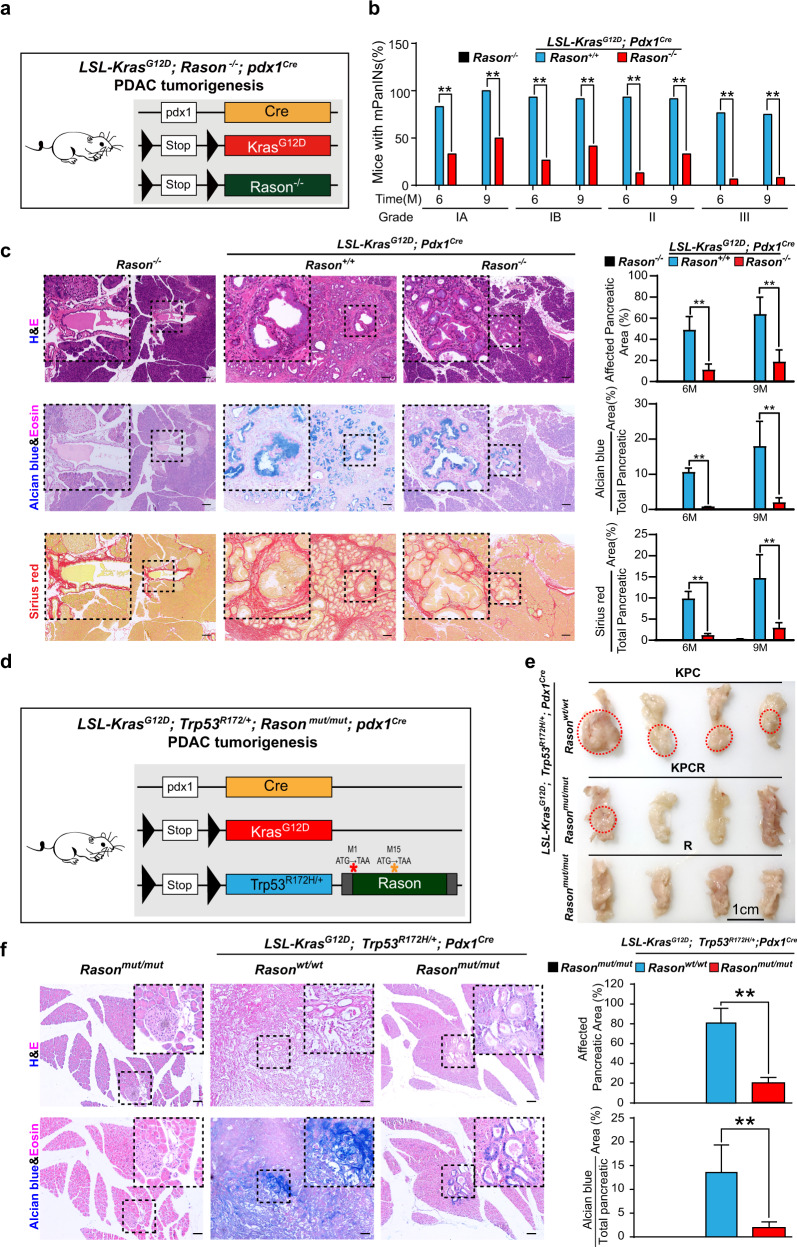
RASON is required for KRAS-driven pancreatic tumorigenesis. **a**–**c** Effect of *Rason* KO on pancreatic intraepithelial neoplasia (PanIN) formation in *LSL-Kras*^*G12D*^; *Pdx1*^Cre^ mice (KC mice). **a** Scheme of establishing *LSL-Kras*^G12D^; *Pdx1*^Cre^; Rason^−/−^ mice (KCR mice). Rason^−/−^ mice was crossed with KC mice to generate KCR mice. **b** *Rason* KO inhibited PanIN formation in KC mice. Both KC and KCR mice were grown to 6 or 9 months old and the percentage of mice with different PanIN grades were shown for each group (*n*  = 30 mice). **c** Histopathologic lesions observed in the pancreas of mice from three different groups (*Rason*^−^/(R), *LSL-Kras*^*G12D*^; *Pdx1*^Cre^; *Rason*^−/−^ (KCR) and *LSL-Kras*^G12D^; *Pdx1*^Cre^ (KC)) at the age of 6 and 9 months. Representative images (left, 9 months) and quantification (right, 6, and 9 months) of HE (top), Alcian blue (middle), and Sirius red (bottom) staining are shown (*n*  =  30 mice). **d**–**f** Effect of *Rason* KO on PDAC tumor formation in *LSL-Kras*^G12D^; *Trp53*^*R172H/+*^; *Pdx1*^Cre^ mice (KPC mice). Scheme of establishing *LSL-Kras*^G12D^;*Trp53*^R172H/+^; *Pdx1*^Cre^; *Rason*^*mut/mut*^ mice (KPCR mice) (**d**). Two mutations in *Rason* ORF (M1X and M15X) were introduced by CRISPR/Cas9 into the KPC mice to generate KPCR mice. *Rason* KO inhibited PDAC formation in KPC mice (**e**). Mice in all groups (KPC, *n*  = 15; KPCR, *n*  =  10; R (*Rason*^mut/mut^), *n* = 10) were sacrificed at 3 months of age. Representative images of mouse pancreas from each group are shown; red circles indicate pancreatic tumor. Histopathologic analysis of mouse pancreas from each group (**f**). Top, HE staining; bottom, Alcian blue staining; left, representative images (bars, 100  µm); right, quantification. Data shown are means  ±  SD. *P* values were calculated by paired Student’s *t*-test (**b**, **c**, **f**). **P  <  0.01.

To further test the role of RASON in tumorigenesis in vivo, we first crossed *Rason*^*−/−*^ mice with *LSL-Kras*^*G12D*^*; Pdx1*^*Cre*^ mice (KC mice)^25^ to generate *LSL-Kras*^*G12D*^*; Pdx1*^*Cre*^*; Rason*^−/−^ mice (KCR mice; Fig. 4a; Supplementary information, Fig. S9). The KC model is a well-established model for studying early stages of PDAC development, i.e., the formation of pancreatic intraepithelial neoplasia (PanIN). Compared with KC mice, KCR mice exhibited a significant reduction in the formation of PanIN lesions of all grades (IA, IB, II, III) at both 6 and 9 months of age (Fig. 4b, c), suggesting RASON is involved in the early stage of KRAS-driven pancreatic tumor initiation. Next, to assess the role of RASON in PDAC formation in the classical *LSL-Kras*^*G12D*^*; Trp53*^*R172H/+*^*; Pdx1*^*Cre*^ model (KPC mice), we introduced *Rason* KO into the KPC background by CRISPR/Cas9-mediated mutation of the start codon in *Rason* ORF (Fig. 4d; Supplementary information, S10; natural breeding was not feasible because of the proximity of *Rason* and *p53* gene allele on the same chromosome) and generated *LSL-Kras*^*G12D*^*; Trp53*^*R172H/+*^*; Pdx1*^*Cre*^*; Rason*^*mut/mut*^ mice (KPCR mice). KPC mice develop high-stage PDAC in 3–6 months. Compared to KPC mice, KPCR mice exhibited a significant reduction in both tumor incidence (6/15 in KPC vs 2/10 in KPCR, 3 months) and tumor size (Fig. 4e, f). Notably, as a byproduct of KPCR mice, *Rason*^*mut/mut*^ mice (with mutations in the start codon of *Rason* ORF) showed a significant reduction in KRAS signaling in primary MEF cells (Supplementary information, Fig. S10b–d), similar to that observed in *Rason*^*−/−*^ mice (Fig. 2h). These data showed that RASON is required for KRAS-driven pancreatic tumorigenesis in mice.

**Fig. 5:**
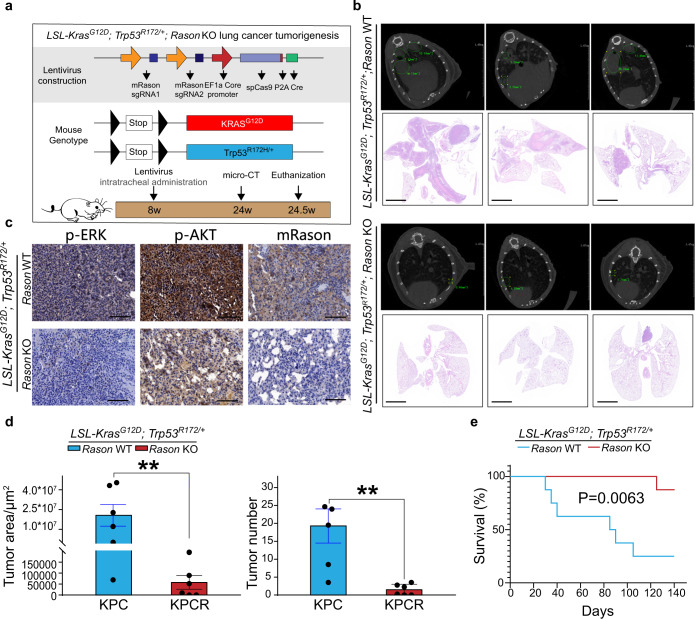
RASON is required for KRAS-driven lung tumorigenesis. **a** Schematic diagram of *Rason* KO in *LSL-Kras*^*G12D*^*; Trp53*^*R172H/+*^ (KP) mice. Both *Kras* and *p53* mutations are activated in the lung by intratracheal administration of Cre. To achieve simultaneous KO of *Rason*, a lentivirus containing Cre, Cas9, and two Rason sgRNAs were used. **b**–**e** *Rason* KO in KP mice significantly inhibited lung tumor formation. Representative images showing micro-CT and HE staining of lung tissues derived from KP mice with or without *Rason* KO (bars, 2 mm) (**b**). Immunohistochemistry showing the status of KRAS effector signaling in lung tumor tissue from KP mice with or without *Rason* KO (**c**). Quantification of lung tumor area and tumor numbers from KP mice with or without *Rason* KO (**d**). Kaplan-Meier curve showing overall survival of KP mice with or without *Rason* KO (**e**). Data are shown as means ± SEM. *P* values were calculated by paired Student’s *t*-test (**d**) and log-rank analysis test (**e**). **P < 0.01.

Another genetic mouse model that is often used for investigating KRAS-driven tumorigenesis is the *LSL-Kras*^*G12D*^*; Trp53*^*R172H/+*^ (KP mice) lung cancer model. In this model, simultaneous KO of *Rason* and activation of *Kras/p53* alleles in the lung was achieved by intratracheal administration of a lentivirus construct containing Cre, Cas9, and *Rason* sgRNA (Fig. 5a). *Rason* KO markedly inhibited lung tumor formation in KP mice, as monitored by micro-CT measurement of lung tumor incidence and size (Fig. 5b–d), and significantly prolonged mouse survival (Fig. 5e), suggesting a common mechanism underlying the protumor function of RASON in KRAS mutant cancers. Altogether, the above results demonstrated an essential role for RASON in KRAS-driven tumorigenesis in vitro and in vivo.

### RASON directly binds to KRAS

**Fig. 6:**
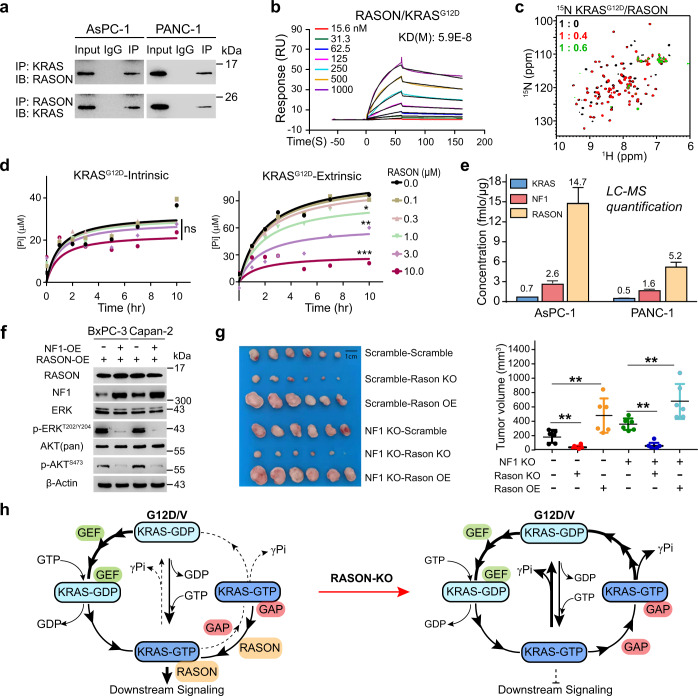
RASON directly binds to KRAS and stabilizes KRAS in the GTP-bound hyperactive state. **a** IP showing the mutual binding between RASON and KRAS in PDAC cells. **b** SPR analysis of the interaction between RASON and KRAS^G12D^. **c** 2D ^1^H-^15^N HSQC spectra of ^15^N-labeled human KRAS in the absence and presence of human RASON. ^15^N-labeled KRAS^G12D^ was mixed with unlabeled RASON at the molar ratios of 1:0.4 (red) and 1:0.6 (green), respectively. The peak intensities of KRAS residues decreased significantly in the presence of RASON, indicating a strong interaction between KRAS and RASON. **d** Intrinsic (left) and NF1-stimulated (right) KRAS^G12D^ GTPase activity in the presence of RASON were measured using Malachite Green assay (RASON was titrated in 3-fold dilution series). **e** Quantification of KRAS, NF1, and RASON proteins in PDAC cells using LC-MS. **f** IB of KRAS effector signaling in BxPC-3 and Capan-2 RASON-OE cells with forced NF1 overexpression. **g** Effect of *Nf1* KO plus *Rason* KO/OE on the tumor formation of KRAS^G12D^ MEF cells in nude mice. **h** Schematic diagram of RASON regulation of oncogenic KRAS signaling. In KRAS mutant cancers, RASON is overexpressed and competes with GAPs (e.g., NF1) for KRAS binding. RASON binding to KRAS impairs both intrinsic and extrinsic GTP hydrolysis of mutant KRAS and trapped it in the GTP-bound state, resulting in continuous KRAS activation. In the absence of RASON (e.g., KO), GAPs induce GTP hydrolysis and shift the KRAS nucleotide cycle towards the GDP-bound state, resulting in inhibition of KRAS downstream signaling. Data shown are means ± SD. *P* values were calculated by two-way ANOVA test (**d**) and one-way ANOVA test (**g**). *P < 0.05, **P < 0.01, ***P < 0.001.

To investigate how RASON affects KRAS activity, we first screened for RASON binding partners using immunoprecipitation (IP)-MS in AsPC-1 (KRAS^G12D^) cells. KRAS and RAS-GAPs (IQGAP1/2, NF1, RASA2) were among the top hits identified (Supplementary information, Fig. S11a). Conversely, RASON peptide signal could also be identified in IP-MS experiments in AsPC-1 and PANC-1 cells using anti-KRAS antibody (Supplementary information, Fig. S11b, c). The mutual binding between RASON and KRAS was then confirmed by two-way IP in PDAC cells (Fig. 6a). Interestingly, RASON did not co-IP with other RAS family members such as Rho and CDC42 (Supplementary information, Fig. S11d), suggesting its specificity for RAS. Confocal microscopy showed that RASON co-localizes with KRAS in MEF cells (Supplementary information, Fig. S12a), PDAC cell lines (Supplementary information, Fig. S12b), and PDAC patient tissues (Supplementary information, Fig. S13a), further supporting the direct interaction between RASON and KRAS.

We next tested the binding between purified RASON protein and different KRAS mutants using Surface Plasma Resonance (SPR). RASON binds to KRAS^G12D^ and KRAS^G12V^ with remarkably high affinity (*K*_d_ = 5.9E–8 M and 1.0E−7 M, respectively, Fig. 6b; Supplementary information, Fig. S14a). Notably, the *K*_d_ value for KRAS–RASON binding is within the same order of magnitude as that between KRAS and RAS-GEFs or RAS-GAPs,^26,27^ suggesting that RASON might function as GEF or GAP to interact with KRAS. To further confirm this binding, we performed Nuclear Magnetic Resonance (NMR) titration experiments. ^15^N-labeled KRAS^G12D^ was mixed with unlabeled human RASON protein at molar ratios ranging from 1:0 to 1:0.6, and a dose-dependent decrease of peak intensities were observed in the 2D ^1^H-^15^N heteronuclear single quantum coherence (HSQC) spectra of KRAS in the presence of RASON (Fig. 6c), suggesting a strong interaction between human RASON and KRAS^G12D^. Taken together, SPR and NMR data strongly support the direct binding between KRAS and RASON.

### RASON inhibits GAP-induced GTP hydrolysis and maintains KRAS in the active state

In the classical KRAS regulation model, RAS-GEFs activate KRAS by promoting GDP-GTP exchange, while RAS-GAPs deactivate KRAS by catalyzing GTP hydrolysis; mutations in the G12 position partially inhibit GAP-mediated GTP hydrolysis, thus keeping KRAS in the active state. Since RASON promotes oncogenic KRAS signaling, we first tested the possibility of RASON being a new GEF for KRAS by performing in vitro nucleotide exchange assays. Surprisingly, however, RASON had no effect on the rate of either intrinsic or GEF-induced GDP-GTP exchange for KRAS^G12D^ or KRAS^G12V^, suggesting that it is not a RAS-GEF (Supplementary information, Fig. S14b). To test the other side of the coin, i.e., RASON being an inhibitor of GAP activity, we carried out in vitro GTP hydrolysis experiments. As a result of the G12 mutations, KRAS^G12D^ and KRAS^G12V^ proteins exhibited a very slow rate of intrinsic GTP hydrolysis but still retained certain GAP-induced extrinsic GTP hydrolysis activity. However, the presence of RASON almost completely diminished the hydrolysis capability induced by GAP (Fig. 6d; Supplementary information, Fig. S14c; NF1 was used as the representative RAS-GAP here). These data demonstrated that RASON could inhibit GAP-induced KRAS-GTP hydrolysis in cell-free systems. Notably, RASON could also suppress, but to a lesser extent, extrinsic GTP hydrolysis activity in KRAS^WT^ protein (Supplementary information, Fig. S14d); however, KD of RASON in BxPC-3 cells (KRAS^WT^) did not affect its proliferation (Supplementary information, Fig. S14e), suggesting that the physiological function of KRAS^WT^ does not depend on RASON signaling.

We next tested if RASON could also compete with GAP for KRAS binding and inhibit GAP-induced GTP hydrolysis in cancer cells.^28,29^ IP experiments in HEK293T cells showed that increasing levels of RASON overexpression dose-dependently inhibited KRAS-NF1 binding (Supplementary information, Fig. S15a), suggesting that RASON can compete with NF1 (as the representative GAP) in cells. A prerequisite for this hypothesis is that RASON is expressed in cells at a sufficiently high level to bind KRAS protein and to compete against RASGAPs. To evaluate the abundance of RASON in cells, we used MS to quantify the respective protein levels of RASON, KRAS and NF1 in AsPC-1 and PANC-1 cells. Purified RASON, KRAS and NF1 proteins were first used to develop MS methods and generate a standard curve for each protein (Supplementary information, Fig. S16). Intracellular levels of each protein were then determined by detection and quantification of the respective signature peptides according to the standard curve. Cellular levels of RASON protein were found to be 10–20 folds higher than that of KRAS and 3–5 folds higher than NF1 (Fig. 6e), suggesting that RASON is expressed at a sufficiently high level to bind to KRAS and competes with NF1 in PDAC cells. Moreover, overexpression of NF1 in BxPC-3 and Capan-2 RASON-OE cells abolished the activation of KRAS signaling induced by RASON overexpression, further supporting our hypothesis (Fig. 6f). Next, we performed genetic epistasis experiments in the KRAS^G12D^ MEF cell line and compared the effect of *Rason* KO or OE on malignant transformation in the presence or absence of *Nf1* (Fig. 6g; Supplementary information, Fig. S17). *Nf1* KO itself promoted tumor formation, which is consistent with its tumor suppressor role. *Rason* KO decreased and *Rason* OE increased tumor formation in either the *Nf1* WT or *Nf1* KO background. Concomitant *Nf1* KO and *Rason* OE maximumly increased KRAS^G12D^-induced MEF tumor formation (Fig. 6g; Supplementary information, Fig. S17). These results are consistent with our hypothesis that RASON inhibits NF1-induced KRAS deactivation and maintains KRAS in a hyperactive state. Furthermore, RASON could also compete with another RAS-GAP, RASA2 (Supplementary information, Fig. S15b, c) for binding KRAS, implying a common biochemical mechanism. Taken together, these data demonstrated that RASON is a high-affinity KRAS binding protein that prevents both intrinsic and GAP-induced GTP hydrolysis, thus sustaining the hyperactive KRAS phenotype; a graphic model of KRAS regulation by RASON is shown in Fig. 6h.

### RASON is a potential therapeutic target for PDAC

**Fig. 7:**
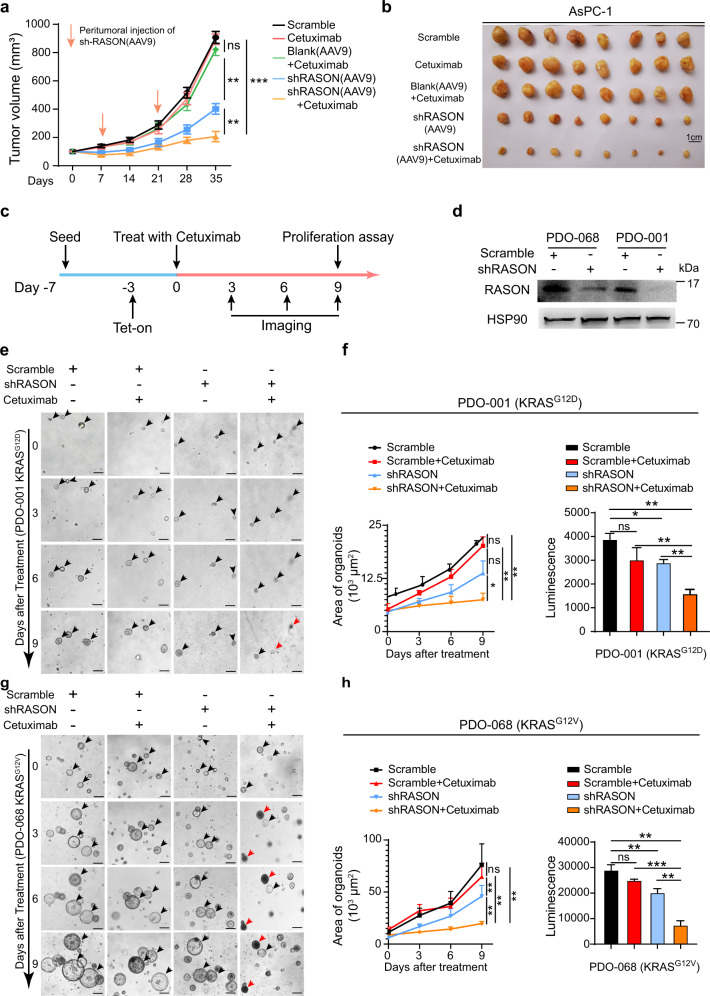
RASON is a potential target for PDAC therapy. **a**, **b** Synergetic effect of RASON KD and cetuximab treatment on AsPC-1 xenograft tumor growth (*n* = 8 mice). AsPC-1 xenograft tumors were treated with either cetuximab, RASON shRNA (AAV9), or both. **c**–**h** Synergetic effect of RASON KD and cetuximab treatment on the growth of human PDAC PDOs. The schematic diagram of experimental design (**c**). KRAS^G12D^ mutant PDO-001 organoids and KRAS^G12V^ mutant PDO-068 organoids stably expressing tetracycline-inducible RASON shRNA were seeded seven days before cetuximab treatment; shRNA KD of RASON was activated three days before treatment. IB showing successful RASON KD in human PDAC PDOs (**d**). Representative images of PDO-001 (KRAS^G12D^) treated with cetuximab, RASON shRNA, or both (bars, 200 µm) (**e**). **f** Growth curves of PDO-001 as measured by organoid area at each time point (left). Organoid growth was also measured by the Cell Titer-Glo assay at the end of experiment (right). Representative images of PDO-068 (KRAS^G12V^) treated with cetuximab, RASON shRNA, or both (bars, 200 µm) (**g**). **h** Growth curves of PDO-068 as measured by organoid area at each time point (left). Organoid growth was also measured by the Cell Titer-Glo assay at the end of experiment (right). Data are shown as means ± SEM. *P* values were calculated by two-way ANOVA test (**a**, **f** (left), **h** (left)) and one-way ANOVA test (**f** (right), **h** (right)). **P < 0.01, ***P < 0.001.

Given the critical role of RASON in promoting oncogenic KRAS signaling, we investigated whether this could be utilized for targeting KRAS-driven cancers. Most PDAC patients are resistant to EGFR inhibitors because of KRAS mutations.^30^ We tested the effect of cetuximab, an anti-EGFR antibody that showed no clinical benefits in KRAS mutant cancers, on the growth of two KRAS mutant PDAC cell lines AsPC-1 and PANC-1. As expected, both cell lines were resistant to cetuximab treatment. In sharp contrast, shRNA KD of RASON in these cells resulted in significant growth inhibition and the combination of cetuximab with shRASON maximally blocked cell growth in vitro (Supplementary information, Fig. S18a). In vivo subcutaneous experiments also showed that cetuximab itself had no effect on AsPC-1 tumor growth, whereas AAV9 delivery of RASON shRNA significantly inhibited xenograft tumor growth. Combination therapy of cetuximab and AAV9-delivered RASON shRNA maximally diminished tumor growth in nude mice (Fig. 7a, b; Supplementary information, Fig. S18b). IB and IHC analysis confirmed target engagement (Supplementary information, Fig. S18c, d), demonstrating that RASON inhibition sensitized KRAS mutant cancer cells to cetuximab through impairment of KRAS activation.

Lastly, we tested the effect of combination therapy with cetuximab and RASON shRNA in PDAC patient-derived organoid (PDO) models (Fig. 7c). Two PDOs established in our lab, namely PDO-001 (KRAS^G12D^) and PDO-068 (KRAS^G12V^), were first stably transfected with tetracycline-inducible RASON shRNA constructs. PDOs were then treated with cetuximab, RASON shRNA, or both. In the combination group, RASON KD was activated three days before cetuximab treatment (Fig. 7d). The growth of organoids was monitored by measuring organoid area on pictures taken at various time points or by measuring the number of viable cells using the Cell Titer-Glo assay at the end of the experiment. Therapeutic responses similar to that in PDAC xenograft experiments were observed in both KRAS^G12D^ and KRAS^G12V^ PDOs (Fig. 7e–h). Collectively, these data indicate that RASON could be a potential therapeutic target for KRAS mutant PDAC patients.

## Discussion

The mechanism of RASON function as we revealed here complements the classical model of RAS regulation (Fig. 6h). Conventional wisdom that KRAS activating mutations (G12C, G12D, G12V, G12S, etc.) lose their GTP hydrolysis capability and cannot cycle back to the GDP-bound state has already been challenged by recent evidence.^31^ In fact, these mutants still retain various degrees of both intrinsic and extrinsic GTP hydrolysis capability and could indeed cycle back to their inactive state,^31^ implicating the existence of an additional layer of fine-tuning for RAS regulation. To our knowledge, RASON is the first identified positive regulator of KRAS that is neither GEF nor GAP but directly binds to KRAS and stabilizes it in the hyperactive state. We speculate that RASON does this by competing with RAS-GAPs like NF1 for KRAS binding, thus abolishing the GTP hydrolysis capacity induced by RAS-GAPs. Whether a feedback mechanism exists between RASON and KRAS warrants further investigation. Moreover, our work also puts LINC00673 out of the class of lincRNAs and into the class of more conventional genes. Although divergent functions have been reported for LINC00673 in human cancers,^32^ our data support an oncogenic role of it in PDAC and lung cancer through regulation of KRAS.

Direct targeting of KRAS has been considered ‘mission impossible’ for many years until the recent discovery of small-molecule covalent inhibitors of KRAS^G12C^.^12,33^ KRAS-driven cancers are also resistant to therapies targeting RTKs such as EGFR. Besides, drugs that target KRAS effectors, including sorafenib (RAF inhibitor), rigosertib (RAS mimetic that prevent PI3K interaction) did not achieve desired clinical benefits in patients with KRAS mutation. Due to the critical role of RASON in KRAS-induced tumorigenesis and maintenance, as well as the aberrant overexpression of RASON in many cancers (our unpublished data), targeting RASON, either alone or in combination with other KRAS pathway inhibitors, may provide a novel approach for treating KRAS mutant cancers.

In conclusion, we have discovered a novel protein RASON that is essential for KRAS signaling. Encoded by LINC00673, RASON directly binds to KRAS and stabilizes KRAS in its GTP-bound hyperactive state and is therefore required for KRAS-driven tumorigenesis and tumor maintenance. The therapeutic potential of our findings was confirmed by the sensitization of pancreatic cancer to EGFR inhibitors upon RASON depletion in PDO models. KRAS−RASON interaction, therefore, represents a vulnerable target for the ‘undruggable’ KRAS oncogenic signaling in human cancers.

## Materials and methods

### Human pancreatic cancer specimens

All human cancer tissues were obtained from the Department of Gastrointestinal Surgery and Department of Hepatological surgery, the First Affiliated Hospital of Sun Yat-sen University (IRB: 2017247) or the Department of Surgery, the Affiliated Hospital of Nanjing University (Nanjing Drum Tower Hospital, IRB: 2020-072-01). Tissues were obtained with patients’ written consent under a protocol approved by the institution’s Institutional Review Board and confirmed by a pathologist before use.

### Cell lines and cell culture

All human cell lines were obtained from ATCC (BxPC-3: CRL-1687; Capan-2: HTB-80; Panc02.03: CRL-2553; CFPAC1: CRL-1918; PANC-1: CRL-1469; AsPC-1: CRL-1682; and HEK293T: CRL-3216). Cells were maintained at 37 °C in a humidified atmosphere with 5% CO_2_. Cells were cultured in either RPMI 1640 or Dulbecco’s modified Eagle’s medium (DMEM) according to ATCC instructions with 10% fetal bovine serum (FBS). RAS-less MEF cell lines stably overexpressing KRAS^G12D^ or KRAS^G12V^ were obtained from NIH’s RAS Initiative and cultured as indicated in the instructions online (https://www.cancer.gov/research/key-initiatives/ras/ras-central/blog/2017/rasless-mefs-drug-screens). The in-house primary KRAS^G12D^ MEF cell line was established from E13.5 *LSL-Kras*^*G12D*^ genetic mouse and cultured in DMEM with 10% FBS and 1% GlutaMAX (Thermo Fisher, MA, USA, 35050061). Two additional cell lines were used to produce condition medium for PDAC organoid culture. L Wnt-3A (CRL-2647™) was purchased from ATCC for Wnt3a conditioned medium production and cultured in advanced DMEM/F12 supplemented with 10% FBS. HA-R-Spondin1-Fc 293T Cells (R&D, Minneapolis, USA, 3710-001-01) for producing RSPO-1 conditioned medium was cultured in DMEM supplemented with 10% FBS. All cell lines were regularly tested for mycoplasma contamination.

### Ribosome sequencing and data processing

The ribosomal profiling technique was processed as reported previously,^34^ with a few modifications as described below. Cells or tissues were treated with harringtonine (2 µg/mL, Abcam, Waltham, USA, ab141941) for 2 min and cycloheximide (100 µg/mL, Sigma, MO, USA, 66-81-9) for 3 min. Samples were then dissolved in lysis buffer. Lysates were centrifuged at 20,000× *g* for 10 min at 4 °C, then the supernatant was incubated with 7.5 µL RNase I (NEB, Ipswich, USA, M0307) and 5 µL DNase I (NEB, Ipswich, USA, M0303) in 300 µL total volume for 45 min at room temperature followed by the addition of 10 µL SUPERase·In RNase inhibitor (Ambion, TX, USA, AM2696) to stop nuclease digestion. Size exclusion columns (Illustra MicroSpin S-400 HR Columns, GE Healthcare, 27-5140-01) were equilibrated with 3 mL of polysome buffer by gravity flow and then centrifuged at 600× *g* for 4 min at room temperature. 100 μL of digested RPFs was then added and centrifuged at 600× *g* for 2 min. 10 μL of 10% (wt/vol) SDS was then added and RPFs with size greater than 17 nt were isolated according to the RNA Clean and Concentrator-25 kit (Zymo Research, Irvine, USA, R1017). rRNA was removed using the method reported previously.^35^ In brief, short (50–80 bases) antisense DNA probes complementary to rRNA sequences were added to solution containing RPFs; then RNase H (NEB, Ipswich, USA, H0110) and DNase I was added to digest rRNA and residual DNA probes. Finally, RPFs were further purified using magnet beads (Vazyme, Nanjing, China, N412). After obtaining RPFs, Ribo-seq libraries were constructed using NEB Next® Multiple Small RNA Library Prep Set for Illumina® (E7300S, E7300L). In brief, adapters were added to both ends of RPFs, followed by reverse transcription and PCR amplification. The 140–160 bp size PCR products were enriched to generate a cDNA library and sequenced using Illumina HiSeq^TM^ X10 by Gene Denovo Biotechnology Co. (Guangzhou, China).

### RNA sequencing and data processing

Total RNA was first extracted using Trizol reagent kit (Invitrogen, Carlsbad, CA, USA, 15596026) according to the manufacturer’s protocol. Eukaryotic mRNA was then enriched by Oligo(dT) beads and fragmented into short fragments using fragmentation buffer and reverse transcribed into cDNA with random primers. Then the cDNA fragments were purified with QiaQuick PCR extraction kit (Qiagen, Germantown, USA, 28104), end-repaired, poly(A) added, and ligated to Illumina sequencing adapters. The ligation products were size selected by agarose gel electrophoresis, PCR amplified, and sequenced using Illumina HiSeq2500 by Gene Denovo Biotechnology Co. (Guangzhou, China).

### Plasmid construction and transfection of HEK293T cells

RASON, NF1, LINC00673, LINC00673-ORF, LINC00673-ORF-ATG-MUT, and KRAS expression plasmids were cloned into the pCDH-CMV-MCS-EF1-GFP + Puro vector (SBI, CA, USA, pCD513B-1). Plasmids were transfected into cells using Lipofectamine 3000® (Thermo Fisher, MA, USA, L3000015) according to the manufacturer’s protocol.

### Stable transfection of cancer cells using lentiviral vectors

Lentiviral vectors expressing human RASON, mouse Rason, sh-RASON, KRAS, sh-KRAS, or sh-LINC00673 were co-transfected with packaging vectors psPAX2 (Addgene, MA, USA, 12260) and pMD2G (Addgene, MA, USA, 12259) into HEK293T cells for lentivirus production using Lipofectamine 2000 in accordance with the manufacturer’s instructions. To establish stable cell lines, cancer cells were transduced using the respective lentivirus in the presence of polybrene (8 mg/mL, Sigma, MO, USA, TR-1003-G). After 72 h recovery, cells were selected with 2 mg/mL puromycin.

### CRISPR-mediated RASON KO in human PDAC cell lines

The target sequences of RASON sgRNA (TTGGATGGAAAGTGGGGAAT) were designed using the http://crispr.mit.edu/ online tool. To produce CRISPR-lentivirus, HEK293T cells seeded in 100 mm plates were transfected with 10 μg lentiCRISPRv2-gRNA or lentiCRISPRv2 control (Addgene, MA, USA, plasmid #52961) plasmids, 5 μg psPAX2 and 2.5 μg PMD2G plasmids using Lipofectamine 3000 according to the manufacturer’s instructions. After incubation for 48–72 h, the supernatants containing lentivirus were harvested and used to infect cells for 4–6 h. Polyclonal KO cell lines were harvested after five days. Monoclonal cell lines with stable KO of target gene were selected over 1−2 months in 96-well plates.

### CRISPR-mediated mouse Rason KO in Ras-less MEF cells

The sgRNA sequences targeting mouse *Rason* (CGCGGATTGTGTGTTTGCGT and GGGTTGGGGTTCCCCTAACG) were cloned into the eSpCas9-2A-GFP (PX458) plasmid by clone EZ. Plasmid amplification was carried out using DH5α competent cells. Ras-less MEF cells were seeded in 100 mm dishes and transfected at approximately 85% confluency. In brief, 30 μg of sgRNA expression plasmids were transfected using Lipofectamine 3000 according to the manufacturer’s protocol. Three days later, GFP-positive cells were sorted into 96-well plates by flow cytometry, single cell clones were picked and validated after 2 weeks.

### CRISPR-mediated NF1 KO in Ras-less MEF cell lines

The sgRNA sequences targeting mouse *NF1* (CCAGGACATCTCCAAGGATG) was cloned into pLentiCRISPR v2. KRAS^G12D^ MEF cells were seeded in 100 mm dishes and transfected at approximately 85% confluency, followed by puromycin selection.

### PCR

DNA was extracted with DNeasy Blood &Tissue Kit (Qiagen, Germantown, USA, 69504) according to the standard procedure, and subjected to PCR assay. Primer sequences are: mouse *Rason* (forward, 5′-CTTCTGTTTCGGGCTGTACG-3′; reverse, 5′-GGCCAATACCCATCTCTCCA-3′). The resulting DNA was evaluated by agarose gel electrophoresis followed by ethidium bromide (Thermo Fisher, MA, USA, 15585011) staining.

### Reverse transcription and real-time (RT) PCR

Total RNA was extracted with TRIZOL and cDNA was obtained by using PrimeScript RT Master Mix (Takara Bio, Kusatsu, Japan, RR036A). The resulting cDNA was then subjected to RT-PCR analysis with SYBR Select Master Mix (Thermo Fisher, MA, USA, A46012) in a StepOnePlus realtime PCR system (Applied Biosystems). Results were normalized to β-Actin mRNA in each sample. Primer sequences are: LINC00673 (forward, 5′-AGTCTGGAGCGCAGAGGACA-3′; reverse, 5′-TCAATCCACGGATGGAGAAGAG-3′), β-Actin (forward, 5′- CATGTACGTTGCTATCCAGGC-3′; reverse, 5′- CTCCTTAATGTCACGCACGAT-3′).

### Antibody generation and immunoblotting

A rabbit monoclonal antibody against the N-terminal 1–14 residues of human RASON protein was obtained by inoculating rabbits with synthesized peptides. The antibody was purified using affinity chromatograph columns. Cell or tissue lysates were separated by 7.5%–17% SDS-PAGE and subjected to western blotting analysis using the following primary antibodies: anti-RASON (This study; 1:500), anti-C-RAF (CST, Danvers, USA, Cat#9422; 1:1000), anti-p-C-RAF (CST Cat#9421; 1:1000), anti-ERK1/2 (CST Cat#4695; 1:1000), anti-p-ERK1/2 (CST Cat#4370; 1:1000), anti-AKT (CST Cat#4691; 1:1000), anti-p-AKT S473 (CST Cat#3787; 1:1000), anti-p-AKT T308 (CST Cat#13038; 1:1000), anti-β-actin (Sigma, MO, USA, Cat#SAB1305567; 1:5000), anti-FLAG (Sigma Cat#SAB1306078; 1:1000), anti-KRAS (Abcam, Waltham, USA, Ab180772; 1:1000), anti-NF1 (Abcam Ab17963; 1:1000), anti-Rho A + B + C (Abcam Ab175328; 1:1000), anti-cdc42 (Abcam Ab187643; 1:1000), anti-ARHGAP10 (Abcam Ab222805; 1:1000). HRP-labeled secondary antibodies of respective species were then used and immunoblot signals were visualized by ECL.

### LC-MS/MS analysis

Total proteins were collected, separated by 17% SDS Gel, stained with Commassie Blue, and the bands at corresponding molecular weight were excised and subjected to trypsin digestion. The resulting peptides were analyzed by QExactive mass spectrometer coupled to a nano-LC (AdvanceLC, Michrom Inc.) The acquired spectra were analyzed with the SEQUEST HT algorithm.

### Immunoprecipitation

HEK293T cells were transfected with different plasmids for 72 h. Cells were lysed in ice-cold lysis buffer (0.3% CHAPS, 10 mM β-glycerol phosphate, 10 mM pyrophosphate, 40 mM HEPES (pH 7.4), 2.5 mM MgCl_2_ and EDTA-free protease inhibitor). The soluble fractions from cell lysates were immunoprecipitated with primary antibodies against RASON, KRAS, FLAG, NF1, Rho, cdc42, or ARHGAP10 by incubation overnight at 4 °C. Protein A or G beads were then added and incubated for 2 h at room temperate. Immunoprecipitants were washed five times with PBST and subjected to immunoblotting.

### RAS activity assay

GTP-bound RAS (active Ras) was measured using the C-Raf RAS-binding-domain (RBD) pull-down and detection kit (8821, Cell Signaling Technology) as instructed by the manufacturer. In brief, for the negative control, 500 µL cell lysates were treated with 10 µL 0.5 M EDTA, pH 8.0 (for a final concentration of 10 mM) followed by adding 5 µL of 100 mM GDP (for the final concentration of 1 mM). The mixture was incubated at 30 °C for 15 min with constant agitation and the reaction was stopped with 32 µL of 1 M MgCl_2_ (for final concentration of 60 mM). All samples (but not including the Input one) were mixed with GST-Raf-RBD and glutathione resin and incubated at 4 °C for 30 min. Bounded proteins were eluted and analyzed by immunoblot.

### Immunofluorescence

Cultured cells were fixed with 4% formaldehyde for 10−15 min and then blocked with 3% BSA and 0.1% Triton X-100 in PBS for 20 min at room temperature. Immunostaining was performed using the following primary antibodies: anti-RASON (This study), anti-KRAS (Abcam, Waltham, USA, Ab180772). Nuclei were counterstained with DAPI. Images were taken with an Olympus FV1000 confocal microscope (Olympus, Japan) or Leica Inverted Confocal SP8 (Leica, Germany).

### EdU incorporation assay

Edu incorporation into proliferating cells were measured using an EdU assay kit (Ribobio, Guangzhou, China, C10310-1) following the manufacturer’s instructions. In brief, PDAC cells were cultured at 3 × 10^3^ cells per well in 96-well plates in triplicate for 36–48 h. Cells were then exposed to 50 μM EdU for 2 h at 37 °C and washed with PBS three times. Next, cells were fixed with 4% formaldehyde for 30 min at room temperature and washed with PBS three times, followed by treatment with 0.5% Triton X-100 for 10 min at room temperature. After three PBS washes, the cells were added with 100 μL of Apollo® reaction cocktail for 30 min and washed with PBS three times. Then 100 μL Hoechst 33342 (5 mg/mL) was used to stain the cells for 30 min followed by three PBS washes. Representative images were taken with an Olympus fluorescence microscope.

### Cell proliferation assay

The cell counting kit-8 assay (CCK8, Dojodo, Tabaru, Japan, CK04) was performed to measure cell proliferation. Five hundred cells were seeded into each well of 96-well plates and allowed to grow or be treated with drugs for different amounts of time as indicated by each experiment. Cell numbers were determined following the manufacturer’s protocol and absorbance was measured at 450 nm on a microplate reader (Thermo Fisher, MA, USA).

### Colony formation assay

For colony formation assays, 300 cells per well were seeded into 6-well plates and incubated for 2 weeks with complete medium. The colonies were fixed with 4% paraformaldehyde and stained with 0.1% crystal violet and counted under a microscope.

### 3D anchorage-independent growth assay

3D colony formation assay was performed to measure malignant transformation of MEF cells, including Ras-less MEF cell lines and primary KRAS^G12D^ MEF cells. Briefly, 1–5 × 10^3^ Rason-KO or control MEF cells were mixed with 0.6% agarose (Sigma, MO, USA, A9414) and seeded into low attachment 96-well plates (Thermo Fisher, MA, USA, 168136) and cultured in DMEM/F12 supplemented with BSA (Sigma, A9576), EGF (Peprotech, Cranbury, USA, GMP100-15), FGF (Prepotech, 100-26) and insulin (Sigma, I3536). Colonies were photographed and counted in 1−2 weeks.

### Immunohistochemistry

Tumor xenografts or surgical specimen tissue slides were deparaffinized, rehydrated through an alcohol series followed by antigen retrieval with sodium citrate buffer. Tumor sections were blocked with 5% normal goat serum (Vector) with 0.1% Triton X-100 and 3% H_2_O_2_ in PBS for 60 min at room temperature and then incubated with anti-RASON (1:100, this study), anti-p-C-RAF (CST, Danvers, USA, 9421, 1:100), anti-p-ERK (CST, 9121, 1:100), anti-p-AKT (CST, 3787 1:100) overnight. Expression levels of those antigens were then detected by HRP-conjugated DAB.

### Alcian blue and sirius red staining

Alcian blue and Sirius red staining of mouse pancreas tissues were carried out according to published protocols.^36^ The expression of mucin was stained with Alcian blue reagents (IHC WORLD, Woodstock, USA, IW3000) according to manufacturer’s instructions. Briefly, pancreas sections were deparaffinized, hydrated, and immersed in Alcian blue solution for 30 min at room temperature. After washing with distilled water, the slides were counterstained in nuclear fast red or eosin. Strongly acidic mucosubstances will be stained blue. The presence of collagen was stained with Sirius red (IHC WORLD, Woodstock, USA, IW3012). In brief, slides were deparaffinized and hydrated and nuclei were stained with Weigert’s hematoxylin for 10 min. Following 10 min of washing in running tap water, the slides were stained in picro-Sirius Red solution containing 0.1% Sirius Red in saturated aqueous solution of picric acid for 1 h. Collagen-rich tissue is stained red on a pale-yellow background.

### Protein expression and purification

For eukaryotic expression, RASON ORF was cloned into the pcDNA3.1 vector and transfected into HEK293T cells using Sinofection Transfection Reagent (Sino biological Inc. Beijing, China, STF02), and cultured with SMM 293-TI medium (Sino biological Inc. Beijing, China, M293TI-1). Shake flask culture condition: 37 °C, 5% CO_2_, 175 rpm table speed. One week after transfection, the medium was collected and filtered through 0.45 µm filters. The purification process contained the following steps: Step 1, the Ni^2+^ packed column was washed with ddH_2_O and equilibrated with binding buffer (50 mM Tris-HCl, 150 mM NaCl, pH 8.0) at a flow rate of 0.75 mL/min. Step 2, samples were then loaded onto the Ni^2+^ packed column at a flow rate of 0.5 mL/min. Step 3, elution buffer was used to elute unbound proteins and molecules (washed twice with 50 mM imidazole and 100 mM imidazole, respectively). Step 4, RASON protein was obtained highly purified by using elution buffer (300 mM imidazole). Step 5, RASON protein was verified by immunoblotting. Step 6, RASON protein was washed with wash buffer (10 mM Tris-HCl, pH 7.6, 150 mM NaCl) at a flow rate of 0.75 mL/min and kept in −80°C.

For prokaryotic expression, human RASON protein fused with a C-terminal TEV protease cleavage site and Tamavidin 2 protein was incorporated into the pET-30a vector between *Nde*I and *Xho*I endonuclease cleavage sites with a 6× His tag at the C-terminus. The protein was overexpressed in BL21(DE3) bacteria as incubated at 37 °C and induced with 1 mM IPTG. After 4 h of expression, the cells were collected by centrifugation at 4 °C and 4000 rpm for 10 min, and then suspended in the buffer of 50 mM Tris-HCl, pH 8.0, 300 mM NaCl, and 2 mM DTT and lysed by sonication in an ice-water bath. The inclusion bodies were washed twice using the lysis buffer and then dissolved in 8 M urea and loaded onto a nickel column. After removing urea step wisely, TEV protease was added to cleave the recombinant protein at 4 °C overnight on the column. The eluted RASON was further purified by Superdex 75 10/300 GL gel-filtration column (GE Healthcare) and the protein concentration was measured by NanoPhotometer N50.

KRAS coding sequence was synthesized by GenScript (Nanjing, Jiangsu, China) and cloned into the pET-30a (+) vector. KRAS^G12D^ and KRAS^G12V^ plasmids were generated through point mutation by high-fidelity PCR. 6×His-tagged recombinant human KRAS^G12D^ and KRAS^G12V^ proteins (residues 1–169) were produced in *Escherichia coli* (*E. coli*) BL21(DE3) using published protocols.^10^ The GAP-related domain (GRD) of NF1 protein (residues 1198–1530) was cloned into the pET-24d vector and expressed in *E. coli* BL21(DE3) as a recombinant protein with N-terminal 6×His-tag using published protocols.^37^ Ras-specific nucleotide exchange factor SOS^cat^ (residues 564–1049) was cloned into the pET-30a vector and produced in *E. coli* BL21(DE3) as reported.^38^ For NMR titration, ^15^N-labeled KRAS^G12D^ protein was produced in *E. coli* BL21(DE3) which was cultured in M9 medium (^15^NH_4_Cl) with 5 g/L glucose, 2 mM MgSO_4_ and 0.1 mM CaCl_2_, and purified as mentioned above.

### NMR experiment

All NMR experiments were carried out at 298 K on a Bruker AVANCE NEO 800 MHz spectrometer equipped with a 5 mm z-gradient ^1^H&^19^F/^13^C/^15^N TCI cryogenic probe. The 2D ^1^H-^15^N HSQC spectra of ^15^N-labeled KRAS^G12D^ (20 μM) in the absence and presence of unlabeled RASON were obtained to detect the interaction. All NMR spectra were processed and plotted by topspin 4.1.1.

### GTP hydrolysis assay

The malachite green assay was used to measure intrinsic and extrinsic GTP hydrolysis rates of KRAS^G12D^ and KRAS^G12V^ by detecting the production of inorganic phosphates.^39^ Briefly, KRAS^G12D^ or KRAS^G12V^ protein was first loaded with GTP (Sigma, MO, USA, 11140957001) for 2 h at 4 °C. For intrinsic hydrolysis, 7.5 µL GTP-loaded KRAS protein in reaction buffer (10 mM Tris, pH 7.6, 150 mM NaCl, 2 mM MgCl_2_, 0.05% Tween-20) was added to wells of clear flat bottom 384-well plates (JET BIOFIL, Guangzhou, China, TCP011384). Then 0.5 µL mixture including RASON and SOS1 was added to start the reaction and the reaction mixture was allowed to incubate at 37 °C from 0−10 h by starting the 10 h reaction first and then subsequent reactions at each time point. Finally, 2 µL EDTA was added to each well to stop reaction. The final reaction volume in each well is 30 µL. The concentrations of each component in the final mixture were as follows: KRAS (4.0 µM), SOS1 (1.0 µM), GTP (100 µM), EDTA (100 µM), RASON (0.0 µM, 0.1 µM, 0.3 µM, 1.0 µM, 3.0 µM, 10.0 µM). The optical absorption was measured by a Spectramax M4 plate reader at 650 nm (Molecular Device, San Jose, USA) after adding 20 µL malachite green to each well. Samples were measured in duplicates for each experiment.

For extrinsic hydrolysis, the experiment was carried out similarly as described for the intrinsic hydrolysis assay using the same plate set-up and plate reader. In brief, 7.4 µL GTP-loaded KRAS protein in reaction buffer was added to each well. Then 0.6 µL extrinsic mixture including ingredients of intrinsic mixture plus NF1 (RAS-GAP) was added to corresponding wells followed by incubation for specified durations at 37 °C. For extrinsic hydrolysis of KRAS, the duration of incubation was set as follows: 0 s, 30 s, 60 s, 120 s, 300 s, 600 s, 1800 s, 3600 s, respectively. Finally, 2 µL EDTA was added to stop reaction. The concentrations of each component in the final mixture were the same as the intrinsic hydrolysis assay except for NF1 (1.0 µM). To eliminate contaminating background phosphate signal, we included a control group with all ingredients except KRAS for every corresponding experiment.

### Nucleotide exchange assay

Nucleotide exchange assay for KRAS^G12D^ and KRAS^G12V^ proteins were carried out according to published protocols.^14^ Briefly, KRAS proteins (10 µM) were loaded with 200 µM mant-GDP (Sigma, MO, USA, 69244) in the presence of 2.5 mM EDTA (Sigma, E5134). After incubation for 1 h at room temperature in dark, 10 mM MgCl_2_ was added to terminate the reaction. The proteins were desalted by NAP-5 columns (GE, Chicago, USA, 17085302). 10 µL of each desalted protein was added to low-volume black bottom 384-well plates (Corning, NY, USA, 4514) followed by 5 μL GMPPNP (sigma, G0635). To initiate the nucleotide exchange reaction, 5 μL of different concentrations of RASON (0.0 μM, 0.3 μM, 1.0 μM, 3.0 μM, 10.0 μM final) with (extrinsic) or without (intrinsic) SOS (1 μM final), EDTA (5 mM final), or reaction buffer was added and fluorescence was monitored on a Spectramax M4 plate reader (355 nm excitation, 448 nm emission) for 1 h at 90-s intervals.

### SPR

A Biacore S200 instrument (GE Healthcare, Chicago, USA) was used to detect protein–protein interactions using a direct binding assay format. Prior to activation, the CM5 chip surface was preconditioned using two 50 μL injections each of 10 mM HCl, 50 mM NaOH, 0.1% SDS, and 0.085% H_3_PO_4_ at a flow rate of 100 μL/min. RASON was immobilized on the sensor surface using standard amine coupling. This was achieved by activating the sensor surface using 7 min injections of a mixture of 11.5 mg/mL N-hydroxysuccinimide with 75 mg/mL 1-ethyl-3-(3-dimethylaminopropyl) carbodiimide hydrochloride. Protein immobilization was accomplished using a 10 min injection of protein (30 μg/mL) in 10 mM NaAc, pH 5.0 buffer. Remaining reactive esters were blocked using a 7 min injection of 1 M ethanolamine, pH 8.5, at a flow rate of 10 μL/min. Reference flow cells were prepared without the protein. Denaturation was achieved by a 30 s injection of 10 mM HCl at a flow rate of 30 μL/min. All binding measurements were performed in 0.01 M HEPES, pH 7.4, 0.15 M NaCl, 3 mM EDTA, 0.005% surfactant P20, at a flow rate of 30 μL/min. KRAS^G12D^ or KRAS^G12V^ was injected over the immobilized RASON protein and reference surface with at least 30 s association and dissociation time. Surface regeneration was achieved by dissociation for a time period allowing the response to return to baseline. Control injections of different concentrations of KRAS^G12D^ and KRAS^G12V^ were used to allow monitoring of the functionality of the protein surface. SPR equilibrium binding data, consisting of Req values from several concentration series, were analyzed by fitting a simple 1:1 binding to yield Rmax and *K*_d_ values using Biacore S200 Evaluation Software.

### Quantification of KRAS, NF1 and RASON protein levels in cells

LC-MS was used to quantify the protein levels of KRAS, NF1, and RASON in AsPC-1 and PANC-1 pancreatic cancer cell lines. Purified KRAS, NF1, and RASON proteins were first serial diluted, digested, and analyzed by NanoLC/Triple TOF 5600 (AB SCIEX, Framingham, USA) to generate a standard curve for each protein. Total proteins from cell lysates were separated by 15% SDS-PAGE, stained with Commassie Blue, and the bands at corresponding molecular weight were excised and subjected to trypsin digestion. The resulting peptides were analyzed by LC-MS. The acquired data were analyzed with the Peakviewer 2.0 and Proteinpilot software. Intracellular levels of each protein were then determined by detection and quantification of the respective signature peptides according to the standard curve.

### Mice and animal housing

Athymic (Ncr nu/nu) mice at 6–8 weeks of age were purchased from GemPharmatech Co, Ltd (Nanjing, China). All mice were housed in a SPF facility (5 mice per cage) under a 12-h light-dark cycle with free access to food and water. All experiments using animals were conducted under the Institutional Animal Care and Use Committee (IACUC)-approved protocols at Nanjing University (IACUC-2101008) or Sun Yat-sen University (2021878) in accordance with NIH and institutional guidelines.

### Xenograft studies

#### In vivo PDAC xenograft growth studies

Mice were randomly assigned to experimental groups for all the experiments. For PDAC cell line xenografts, 1 × 10^7^ BXPC-3 cells or 5 × 10^6^ AsPC-1 and PANC-1 cells in 100 μL culture media/Matrigel (4:1) were injected subcutaneously.

#### Subcutaneous tumor formation of MEF cells

Ras-less MEF cells (1.5–2 × 10^6^) with different genetic manipulations were injected subcutaneously into the dorsal flanks of 6-week-old male athymic nude mice, with each mouse bearing one control tumor and one Rason-KO tumor. Mice were euthanized when the larger tumor reached 1000 mm^3^. The tumors were then excised and measured with a caliper in two dimensions.

#### In vivo xenograft treatment studies

In the treatment studies, mice bearing AsPC-1 xenografts were randomly assigned to five groups: control, cetuximab (10 mg/kg, twice a week; MCE, Monmouth Junction, USA, HY-P9905), Blank (AAV9) + cetuximab, shRASON (AAV9) (1 × 10^13^ vg/mL, 100 μL peritumoral injection at day 7 and 21; Vigene Bioscience, Jinan, China), and cetuximab + shRASON (AAV9). Tumors were measured with digital caliper twice a week, and tumor volumes were determined with the formula: (length × width^2^)/2 and plotted as means ± SEM.

### CRISPR-mediated generation of *Rason*^−*/*−^ mice

The *Rason*^−*/*−^mouse strain was established using CRISPR-mediated deletion of the ORF of mouse Rason. The target sequences of gRNA were: gRNA1: CGCGGATTGTGTGTTTGCGT; gRNA2: GGGTTGGGGTTCCCCTAACG. The Rason-KO allele was bred into C57BL/6 background; and animals were maintained and crossed using standard procedures (Cyagen Biosciences, Guangzhou, China). The following primers were used to identify the homozygous KO mice (forward: 5′-CAACTCCCAGGATACCATTCGGC-3′; reverse: 5′-GAGCCCAGAACAGCCGCTGAC-3′).

### Genetic mouse models for pancreatic tumorigenesis

The *LSL-Kras*^*G12D*^*; Pdx1*^*Cre*^ (KC) genetic mouse strain described previously^40,41^ was provided by Shanghai Model Organisms Center, Inc (Shanghai, China) and crossed with *Rason*^*−/−*^ mice to generate *LSL-Kras*^*G12D*^*; Pdx1*^*Cre*^*; Rason*^−*/*−^ (KCR) mice.

The *LSL-Kras*^*G12D*^*; Trp53*^*R172H/+*^*; Pdx1*^*Cre*^*; Rason*^*mut/mut*^ (KPCR) mouse strain was generated via in vitro fertilization (IVF) by GemPharmatech Co, Ltd (Nanjing, China). In brief, two start codons, the first and the fifteenth ATG, of *Rason* were mutated to stop codon TAA by CRISPR in *LSL-Kras*^*G12D*^*; Trp53*^*R172H/+*^ (KP) mice during the first IVF to obtain *LSL-Kras*^*G12D*^*; Trp53*^*R172H/+*^*; Rason*^*mut/mut*^ (F0) mice, which were used for the second IVF with *Pdx1*^*Cre*^ mice to generate F1 mice. The third IVF was carried out among different genotypes of F2 mice, but only four target mice were obtained from 741 F3 offspring. The fourth IVF was carried out using F3 mice with different genotypes to obtain enough *LSL-Kras*^*G12D*^*; Trp53*^*R172H/+*^*; Pdx1*^*Cre*^*; Rason*^*mut/mut*^ (KPCR) mice. A detailed scheme was also provided in Supplementary information, Fig. S10.

### Genetic mouse model for lung tumorigenesis

The *LSL-Kras*^*G12D*^*; Trp53*^*R172H/+*^ (KP) mice were provided by GemPharmatech, Inc (Nanjing, China). A lentivirus (OBiO Technology, Shanghai, China) containing Cre, two mouse *Rason* sgRNAs (AAAGGCGCAGAGCTGTACG, ACCCACGCGGGGCCGCGGCG), and spCas9 was intratracheally administered at 10^5^ infectious particles to initiate autochthonous lung cancer.^42^ Mice were anaesthetized to evaluate tumor growth by microcomputed tomography (micro-CT) scanning or euthanized to confirm lung adenocarcinoma formation by histological analysis.

### Micro-CT scanning

Micro-CT scans were performed using Hiscan XM Micro CT (Suzhou Hiscan Information Technology, Suzhou, China). The X-Ray tube settings were 60 kV and 133 uA and images were acquired at 50 µm resolution. A 0.5° rotation step through a 360° angular range with 50 ms exposure per step was used. The images were reconstructed with Hiscan Reconstruct software (Version 3.0) and analyzed with Hiscan Analyzer software (Version 3.0).

### Human pancreatic tumor organoid culture and drug treatment

Human PDAC PDOs were established according to the protocol established by the Tuveson group.^43^ In brief, freshly collected tumor tissues from PDAC patients undergoing surgery were minced and digested with 5 mg/mL collagenase II (Thermo Fisher, MA, USA, 17101015) and 10 μg/mL DNase1 (Sigma, MO, USA, D5025) in advanced DMEM/F12 supplemented with HEPES (Thermo Fisher, MA, USA, 15630080), Glutamax (Thermo Fisher, 35050061), and Penicillin/Streptomycin (Thermo Fisher, 15140122) at 37 °C for 20 min. Cells were then seeded in growth factor reduced Matrigel (Corning, NY, USA, 356231) and cultured in human complete culture medium [Advanced DMEM/F12 medium supplemented with HEPES (Thermo Fisher, 15630080), Glutamax (Thermo Fisher, 35050061), penicillin/streptomycin (Thermo Fisher, 15140122), B27 (Thermo Fisher, 17504044), Primocin (100 µg/mL, InvivoGen, Toulouse, France, ant-pm-1), N-acetyl-L-cysteine (1.25 mM, Sigma, A9165), Wnt3a-conditioned medium (50% v/v), RSPO1-conditioned medium (10% v/v), recombinant mNoggin protein (0.1 μg/mL, Peprotech, Cranbury, USA, 250-38), human epidermal growth factor (hEGF, 50 ng/mL, Peprotech, GMP100-15), hGastrin (10 nM, R&D, 3006), fibroblast growth factor 10 (FGF10, 100 ng/mL, Prepotech, 100-26), Nicotinamide (10 mM, Sigma, N0636), PEG2 (1 μM, R&D, 2296/10), Y-27632 (10.5 μM, Sigma, Y0503) and A83-01 (0.5 μM, R&D, 2939)].

For tetracycline-inducible KD of RASON, the organoids were first digested into single cells with TrypLE (Thermo Fisher, MA, USA, 12605010) and transfected using lentivirus carrying the shRNA construct. Three days later, puromycin was added for selection. When needed, 1 µg/mL tetracycline (MCE, Monmouth Junction, USA, HY-A0107) was added to induce RASON shRNA (GGGAAGGACTGATCCACATTC) expression.

For combination therapy with cetuximab and RASON shRNA, organoids were seeded into 48-well plates and recovered for seven days before cetuximab treatment. shRASON was activated by tetracycline three days before cetuximab treatment. Organoids were treated with cetuximab (10 µg/mL, MCE, Monmouth Junction, USA, HY-P9905) with or without shRASON for 9 days and imaged every 3 days. At the end of experiment, CellTiter-Glo 3D Reagent (Promega, Madison, USA, G9683) was used to measure cell viability according to the manufacturer’s instructions.

### Statistical analysis

Statistical analyses were performed using GraphPad Prism 8 (GraphPad Software, Inc., La Jolla, CA). The statistical tests performed were paired student’s *t*-test, multiple *t-*test, one-way ANOVA, two-way ANOVA, Wilcoxon test, or log-rank analysis. Sample sizes (*n*) are indicated in the figure legends. Significance was set as *P < 0.05, **P < 0.01, ***P < 0.005 or ****P < 0.0001 for all data.

### Materials availability

All stable and unique reagents generated in this study are available upon reasonable request.

## Supplementary information

Fig. S1

Fig. S2

Fig. S3

Fig. S4

Fig. S5

Fig. S6

Fig. S7

Fig. S8

Fig. S9

Fig. S10

Fig. S11

Fig. S12

Fig. S13

Fig. S14

Fig. S15

Fig. S16

Fig. S17

Fig. S18

## Data Availability

The sequencing data have been deposited in the National Genomics Data Centre (GSA database, https://ngdc.cncb.ac.cn/) under the accession code PRJCA009808.
