# Supplementary material for: A novel protein RASON encoded by a lncRNA controls oncogenic RAS signaling in KRAS mutant cancers
Source: Cell Res. 2022 Oct 14;33(1):30–45. doi: 10.1038/s41422-022-00726-7 (PMC9810732; doi:10.1038/s41422-022-00726-7)
Supplement: Supplementary file 1 — Fig. S1 [file 41422_2022_726_MOESM1_ESM.pdf]

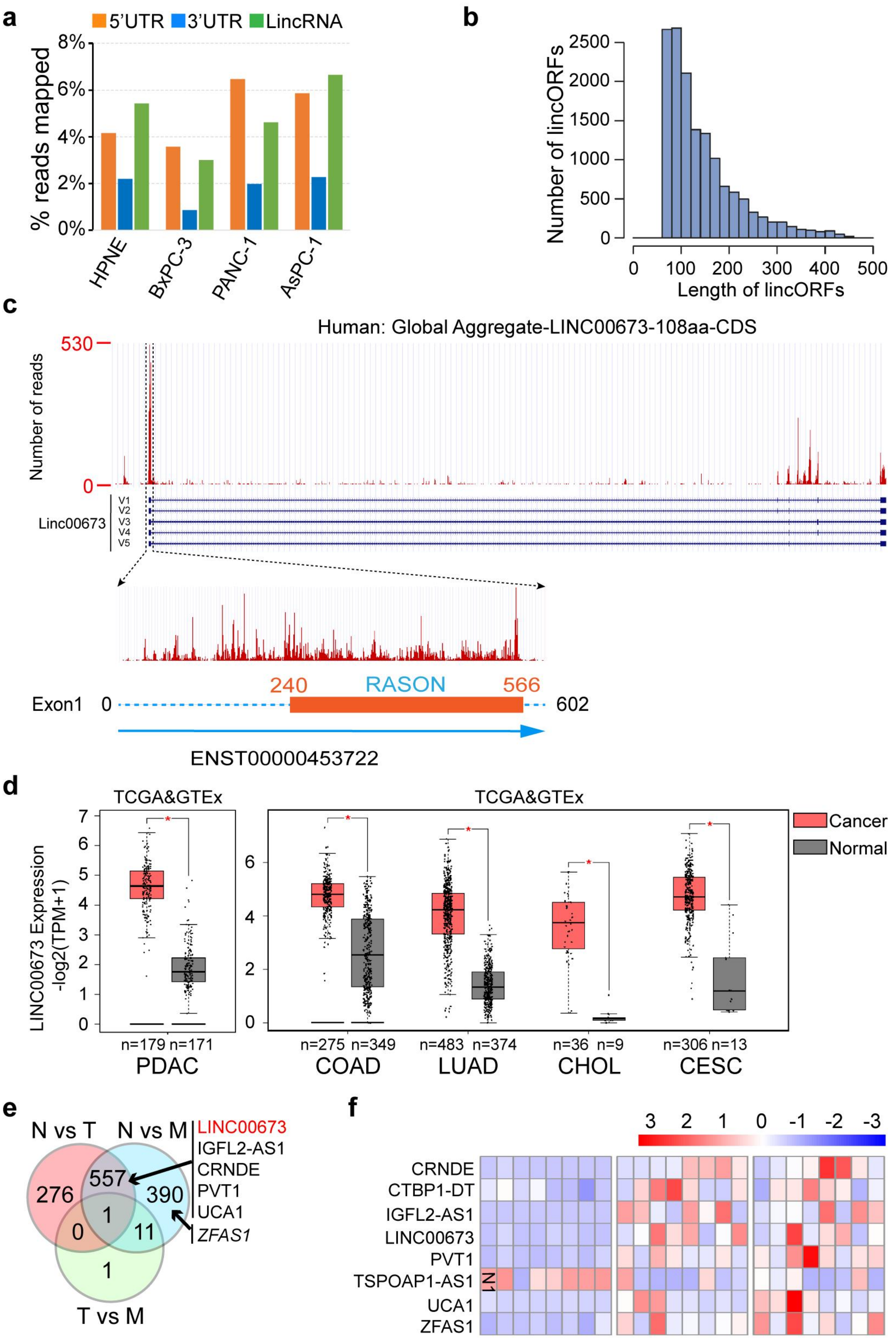

a
5'UTR
3'UTR
LincRNA
% reads mapped
8%
6%
4%
2%
0%
HPNE
BxPC-3
PANC-1
AsPC-1
b
Number of lincORFs
2500
2000
1500
1000
500
0
100
200
300
400
500
Length of lincORFs
c
Human: Global Aggregate-LINC00673-108aa-CDS
530
Number of reads
Linc00673
V1
V2
V3
V4
V5
240
RASON
566
Exon1
602
ENST00000453722
d
TCGA&GTEx
LINC00673 Expression -log2(TPM+1)
n=179 n=171
PDAC
Cancer
Normal
n=275 n=349
COAD
n=483 n=374
LUAD
n=36 n=9
CHOL
n=306 n=13
CESC
e
N vs T
N vs M
T vs M
276
557
390
1
0
11
LINC00673
IGFL2-AS1
CRNDE
PVT1
UCA1
ZFAS1
f
3
2
1
-1
-2
-3
CRNDE
CTBP1-DT
IGFL2-AS1
LINC00673
PVT1
TSPOAP1-AS1
UCA1
ZFAS1
N1

Fig. S1 LINC00673 encodes a novel protein RASON. a Ribosome Protected Fragments (RPFs) map of transcripts annotated as non-coding regions including 5’-UTR, 3’-UTR (of known protein-coding genes) and lincRNAs in HPNE normal pancreas and three PDAC cell lines (BxPC-3, PANC-1, AsPC-1). RPFs are determined by ribosome sequencing. **b** length distribution of the identified lincORFs. X-axis: the length of lincORFs detected in this study, y-axis: the abundance of lincORFs classified by different lengths. **c** active translation of RASON was confirmed in published ribosome profiling datasets. **d** representative cohort from TCGA and GTEx databases showing the expression of LINC00673 RNA in PDAC, colorectal adenocarcinoma (COAD), lung adenocarcinoma (LUAD), cholangiocarcinoma (CHOL) and cervical squamous cell carcinoma (CESC) patients. **e** Venn diagram of differentially expressed lincRNAs among normal (N), tumor (T), and metastasis (M) PDAC paired patient samples by RNA-seq. **f** heatmap showing differential expression of top 8 lincRNAs among tumor (T), adjacent normal tissue (N) and metastatic lymph node (M). Bar graphs show the mean ± SD. *P* values were calculated by one-way ANOVA test (**d**).* $P<0.05$.
