# Supplementary material for: A novel protein RASON encoded by a lncRNA controls oncogenic RAS signaling in KRAS mutant cancers
Source: Cell Res. 2022 Oct 14;33(1):30–45. doi: 10.1038/s41422-022-00726-7 (PMC9810732; doi:10.1038/s41422-022-00726-7)
Supplement: Supplementary file 2 — Fig. S2 [file 41422_2022_726_MOESM2_ESM.pdf]

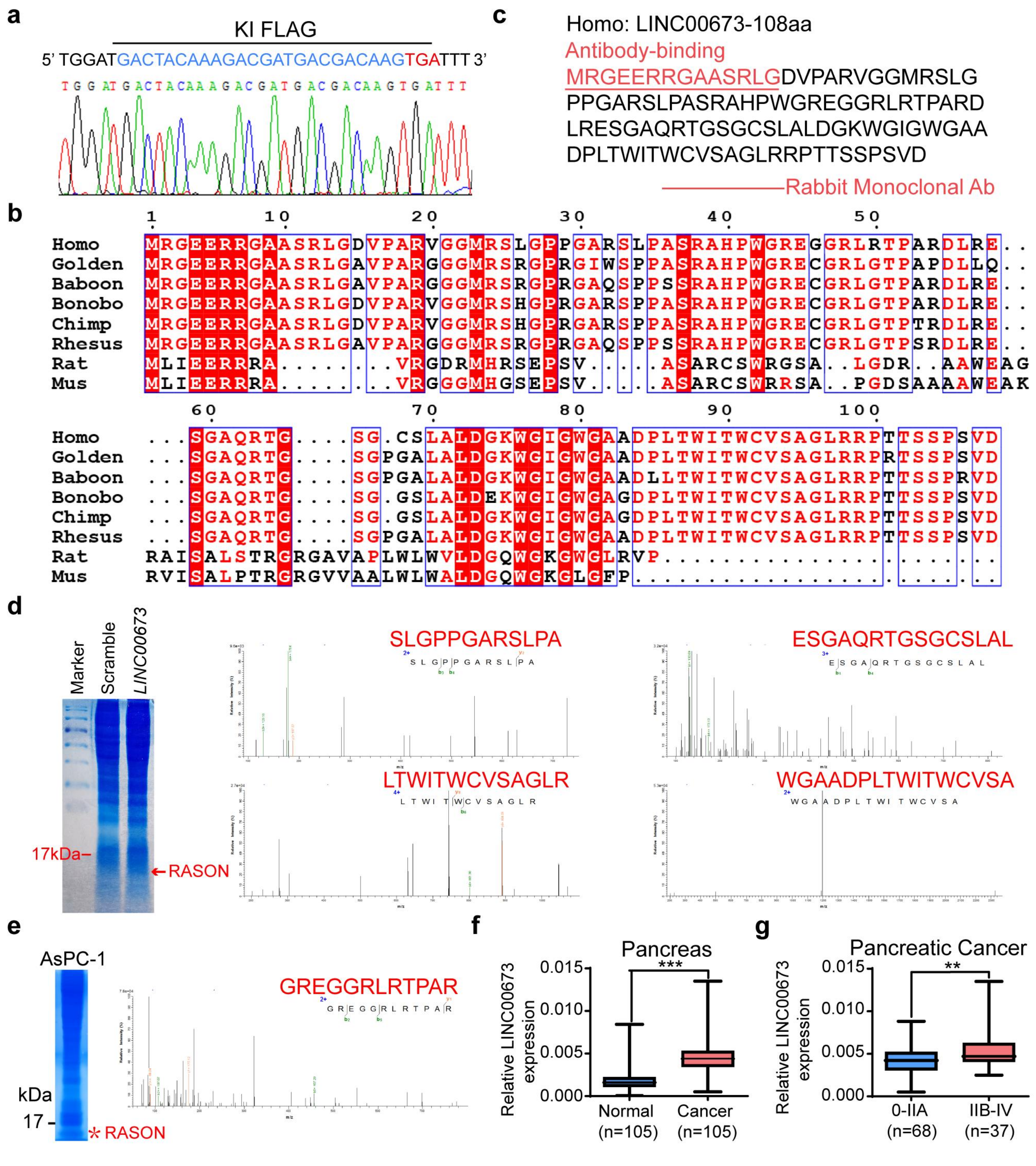

Supplementary information, Fig. S2 Characterization of RASON. a sequencing result showing the successful insertion of FLAG-tag in LINC00673 ORF of HEK293T cells using CRISPR/Cas9. **b** alignment of RASON amino acid sequence among different species. **c** complete amino acid sequence of human RASON, the highlighted N-terminal peptide was used to produce RASON rabbit monoclonal antibody. **d, e** MS detection of RASON-specific peptide sequences from HEK293T cells transfected with LNC00673 (**d**), or endogenously in AsPC-1 cells (**e**). **f** LINC00673 RNA levels in tumor tissues and paired normal tissues from an independent cohort of 105 patients. **g** LINC00673 RNA levels in low grade PDAC (0-IIA) and high grade PDAC (IIB-V) patients. Data in bar graphs are shown as mean ± SD. *P* values were calculated by Wilcoxon test (**f**, **g**). ** $P<0.01$, *** $P<0.001$.
