# Supplementary material for: A novel protein RASON encoded by a lncRNA controls oncogenic RAS signaling in KRAS mutant cancers
Source: Cell Res. 2022 Oct 14;33(1):30–45. doi: 10.1038/s41422-022-00726-7 (PMC9810732; doi:10.1038/s41422-022-00726-7)
Supplement: Supplementary file 3 — Fig. S3 [file 41422_2022_726_MOESM3_ESM.pdf]

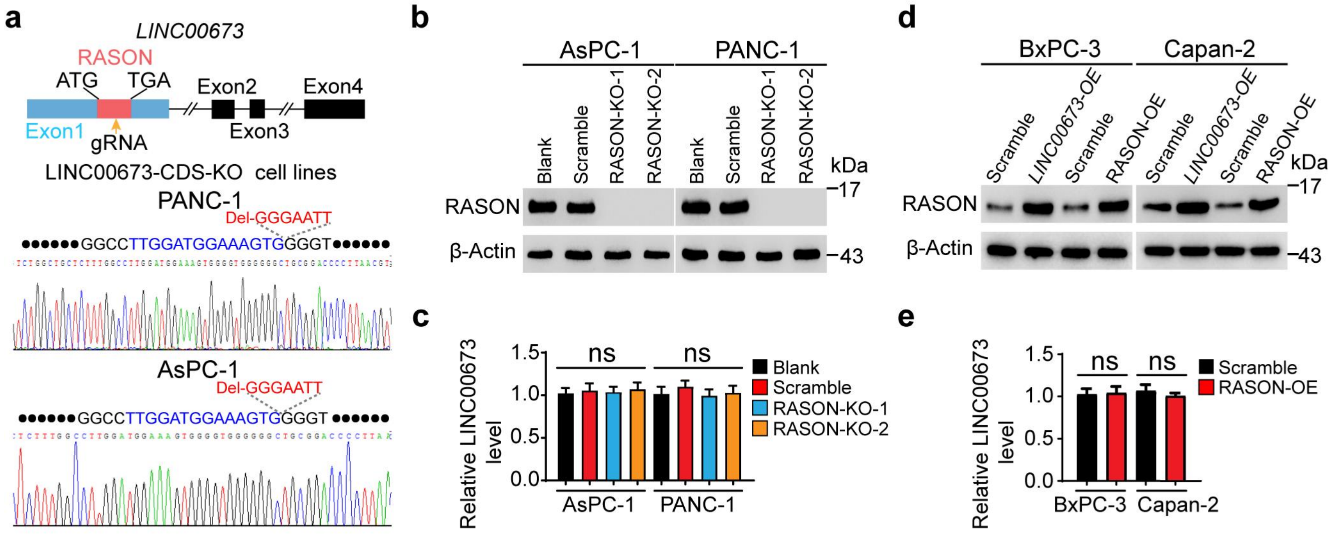

Supplementary information, Fig. S3 Overexpression (OE) and knockout (KO) of RASON in PDAC cell lines. a sequencing results confirming the indel of RASON ORF in AsPC-1 and PANC-1 cells by CRISPR/Cas9. **b, c** RASON protein expression (**b**) and LINC00673 RNA levels (**c**) in RASON KO AsPC-1 and PANC-1 cell lines. **d, e** RASON protein expression (**d**) and LINC00673 RNA levels (**e**) in LINC00673 or RASON OE BxPC-3 and Capan-2 cells. Bar graphs are shown in mean ± SD. *P* values were calculated by Student's t-test (**c**, **e**). ns, non-significant.
