# Supplementary material for: A novel protein RASON encoded by a lncRNA controls oncogenic RAS signaling in KRAS mutant cancers
Source: Cell Res. 2022 Oct 14;33(1):30–45. doi: 10.1038/s41422-022-00726-7 (PMC9810732; doi:10.1038/s41422-022-00726-7)
Supplement: Supplementary file 4 — Fig. S4 [file 41422_2022_726_MOESM4_ESM.pdf]

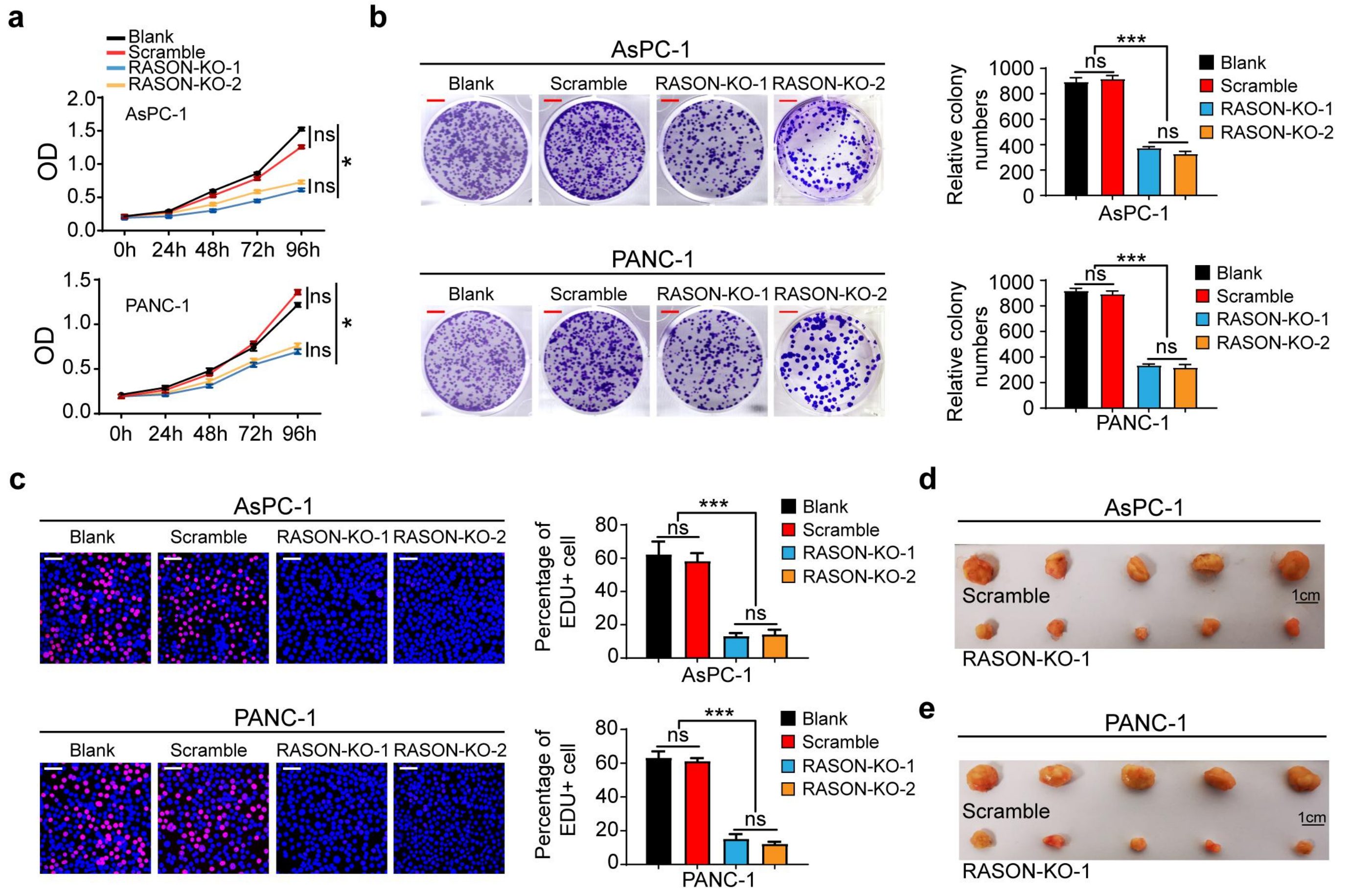

Supplementary information, Fig. S4 Effect of RASON KO on the growth of AsPC-1 and PANC-1 cells *in vitro* and *in vivo*. **a** effect of RASON KO on the proliferation of AsPC-1 and PANC-1 cell lines. **b** effect of RASON KO on the colony formation of AsPC-1 and PANC-1 cell lines. **c** effect of RASON KO on EdU incorporation of AsPC-1 and PANC-1 cell lines (bars, 50 μm). **d, e** Representative tumor images from xenograft experiments using RASON KO AsPC-1 cells **(d)** and PANC-1 cells **(e)**. Data in line and bar graphs are shown as mean ± SD. *P* values were calculated by two-way ANOVA test (**a**) and one-way ANOVA (**b**, **c**). * P<0.05, ** P<0.01, *** P<0.001.
