# Supplementary material for: A novel protein RASON encoded by a lncRNA controls oncogenic RAS signaling in KRAS mutant cancers
Source: Cell Res. 2022 Oct 14;33(1):30–45. doi: 10.1038/s41422-022-00726-7 (PMC9810732; doi:10.1038/s41422-022-00726-7)
Supplement: Supplementary file 5 — Fig. S5 [file 41422_2022_726_MOESM5_ESM.pdf]

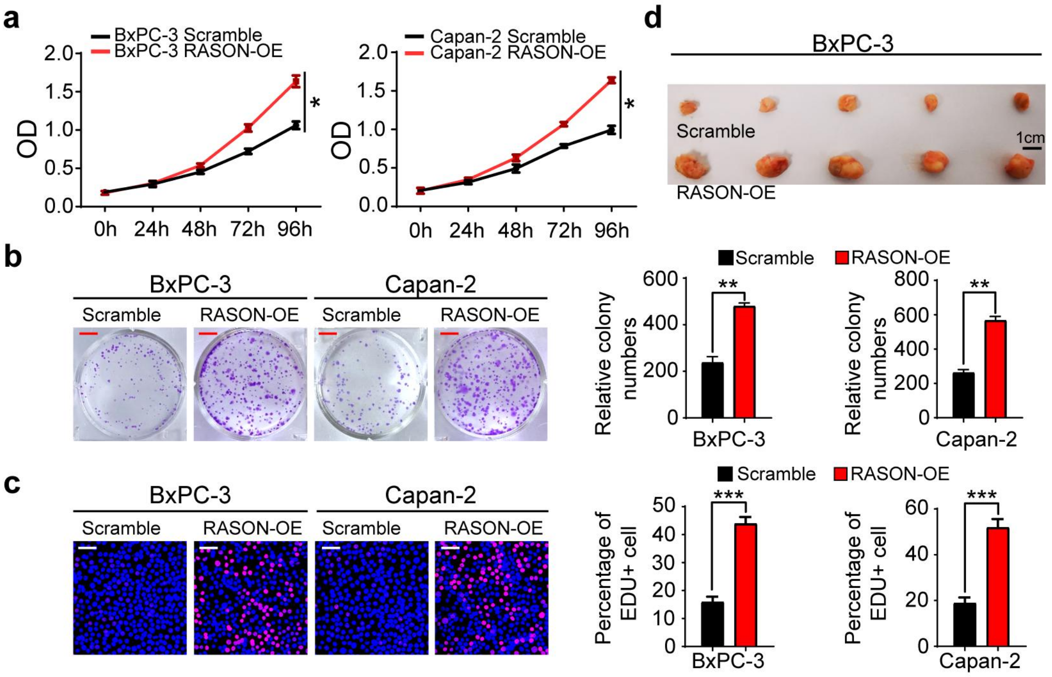

Supplementary information, Fig. S5 Effect of RASON OE on the growth of BxPC-3 and Capan-2 cells *in vitro* and *in vivo*. **a** effect of RASON OE on the proliferation of BxPC-3 and Capan-2 cell lines. **b** effect of RASON OE on the colony formation of BxPC-3 and Capan-2 cell lines. **c** effect of RASON OE on EdU incorporation of BxPC-3 and Capan-2 cell lines (bars, 50 μm). **d** Representative tumor images from xenograft experiments with RASON OE BxPC-3 cells. Data in line and bar graphs are shown as mean ± SD. *P* values were calculated by two-way ANOVA test (**a**) and one-way ANOVA (**b**, **c**). * *P*<0.05, ** *P*<0.01, *** *P*<0.001.
