# Supplementary material for: A novel protein RASON encoded by a lncRNA controls oncogenic RAS signaling in KRAS mutant cancers
Source: Cell Res. 2022 Oct 14;33(1):30–45. doi: 10.1038/s41422-022-00726-7 (PMC9810732; doi:10.1038/s41422-022-00726-7)
Supplement: Supplementary file 6 — Fig. S6 [file 41422_2022_726_MOESM6_ESM.pdf]

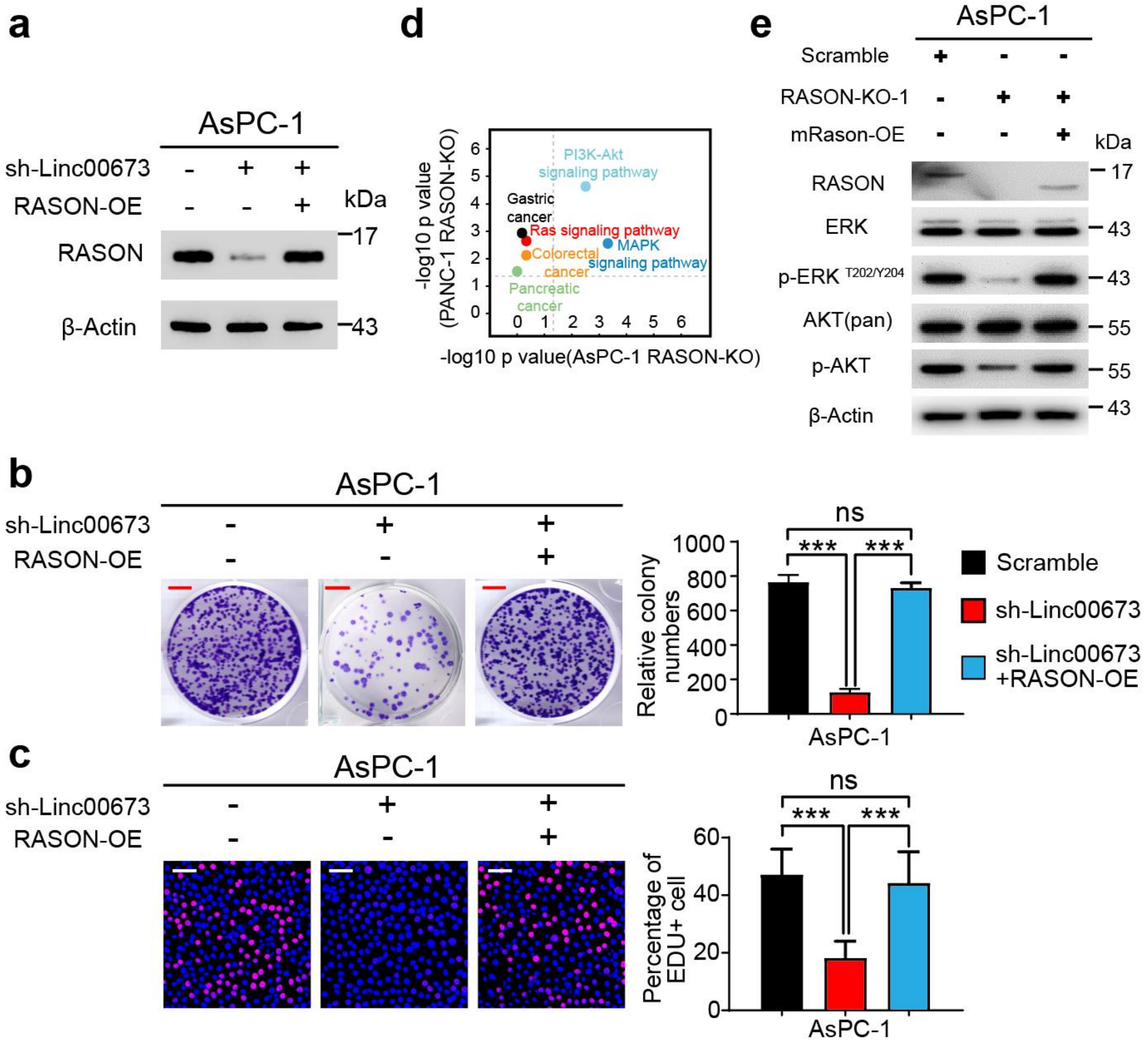

Supplementary information, Fig. S6 RASON promotes the proliferation of PDAC cell lines via KRAS signaling pathway. a-c effect of LINC00673 knockdown (KD) and/or RASON-ORF OE on the proliferation (colony formation assay in **b** and EdU assay in **c**, bars, 50 μm) of AsPC-1 cells. **d** DEG and KEGG analysis of changes in cancer-related pathways in AsPC-1 and PANC-1 RASON KO cells. **e** mouse *Rason* OE rescues human *RASON* KO-induced suppression of RAS signaling. Data in bar graphs are shown as mean ± SD. *P* values were calculated by one-way ANOVA (**b**, **c**). *** *P*<0.001.
