# Supplementary material for: A novel protein RASON encoded by a lncRNA controls oncogenic RAS signaling in KRAS mutant cancers
Source: Cell Res. 2022 Oct 14;33(1):30–45. doi: 10.1038/s41422-022-00726-7 (PMC9810732; doi:10.1038/s41422-022-00726-7)
Supplement: Supplementary file 7 — Fig. S7 [file 41422_2022_726_MOESM7_ESM.pdf]

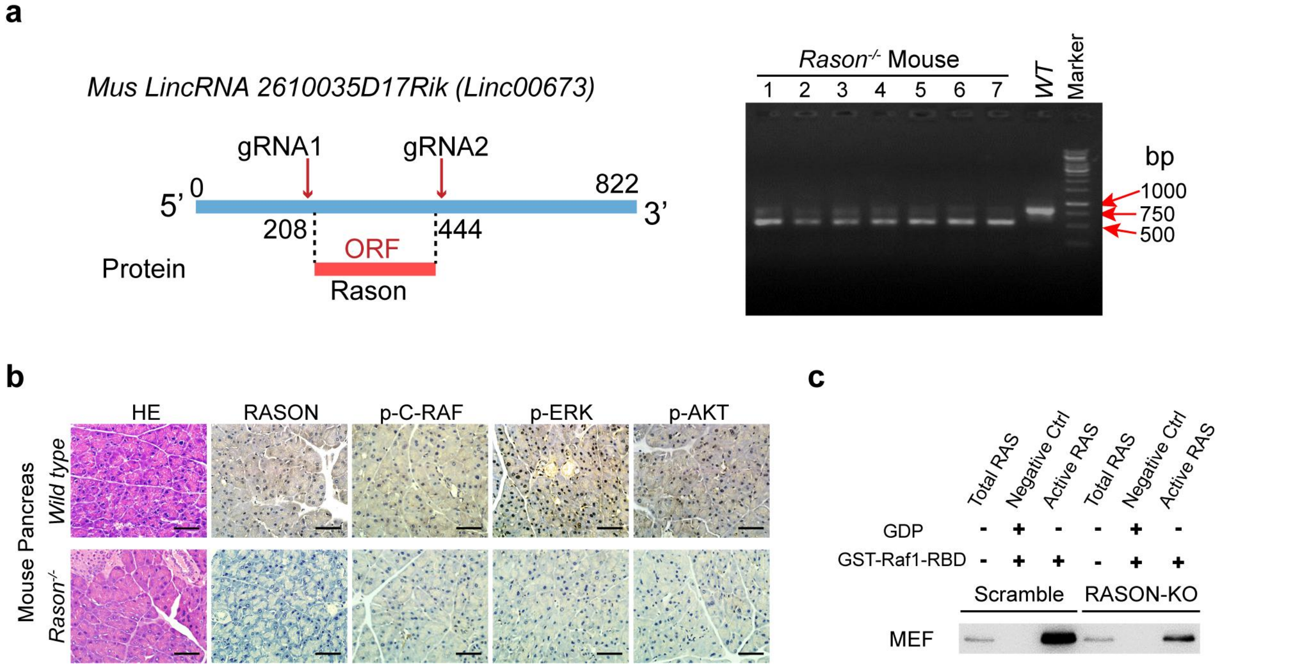

Supplementary information, Fig. S7 Characterization of *Rason* knockout mice. a scheme of homologues *Rason*$^{-/-}$ mice by CRISPR/Cas9 (**left**) and confirmation of *Rason*$^{-/-}$ mice by genotyping (**right**). **b** immunohistochemistry showing the status of KRAS effector signaling in mouse pancreas from wildtype and *Rason*$^{-/-}$ mice (bars, 50 μm). **c** active KRAS-GTP levels as determined by GST-Raf1-RBD pull-down assay in MEFs from wildtype and *Rason*$^{-/-}$ mice.
