# Supplementary material for: A novel protein RASON encoded by a lncRNA controls oncogenic RAS signaling in KRAS mutant cancers
Source: Cell Res. 2022 Oct 14;33(1):30–45. doi: 10.1038/s41422-022-00726-7 (PMC9810732; doi:10.1038/s41422-022-00726-7)
Supplement: Supplementary file 8 — Fig. S8 [file 41422_2022_726_MOESM8_ESM.pdf]

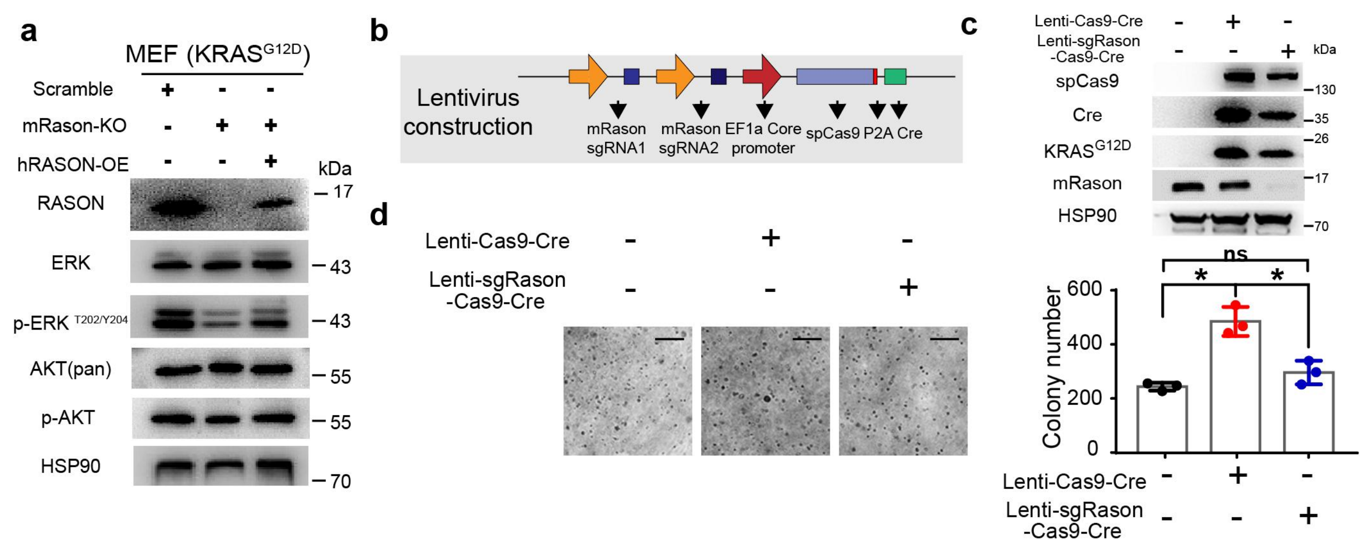


**Supplementary information, Fig. S8 The effect of *Rason* KO on the malignant transformation of MEF cells. a** human RASON rescued mouse *Rason* KO-induced suppression of RAS signaling in KRAS$^{G12D}$ MEF cells. **b-d,** effect of *Rason* KO on the malignant transformation of primary MEF cells derived from the *LSL-Kras$^{G12D}$* mouse strain. **b** schematic of the lentivius structure used to simultaneously activate *Kras$^{G12D}$* mutation and knock out *Rason* in MEF cells derived fome *LSL-Kras$^{G12D}$* mouse. **c** immunoblots showing successful KO of *Rason* and activation of Kras$^{G12D}$ in MEF cells. **d** effect of *Rason* KO on anchorage-independent growth of MEF cells (bars, 1 mm). Data shown are mean ± SD. *P* values were calculated by one-way ANOVA (**d**). * *P*<0.05.
