# Supplementary material for: A novel protein RASON encoded by a lncRNA controls oncogenic RAS signaling in KRAS mutant cancers
Source: Cell Res. 2022 Oct 14;33(1):30–45. doi: 10.1038/s41422-022-00726-7 (PMC9810732; doi:10.1038/s41422-022-00726-7)
Supplement: Supplementary file 9 — Fig. S9 [file 41422_2022_726_MOESM9_ESM.pdf]

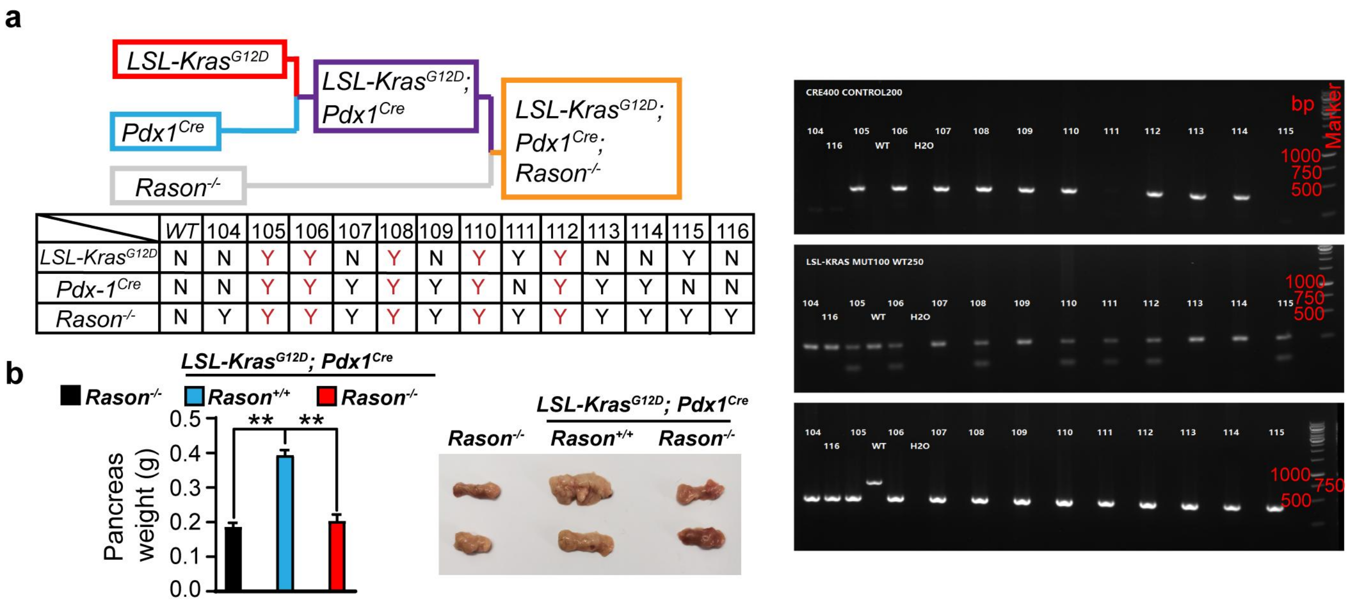

Supplementary information, Fig. S9 Establishment of the *LSL-Kras^{G12D}*; *Pdx1^{Cre}*; *Rason$^{-/-}$* mouse strain (KCR mice). a scheme of establishing *LSL-Kras^{G12D}*; *Pdx1^{Cre}*; *Rason$^{-/-}$* mice (KCR mice) using *LSL-Kras^{G12D}*; *Pdx1^{Cre}* (KC mice) and *Rason$^{-/-}$* mice, and the confirmation by genotyping. **b** effect of *Rason* KO on panIN formation in KC mice. Shown are the pancreas weight of *Rason$^{-/-}$*, KCR, and KC mice at the age of 6 months and representative images showing pancreas size of each group (n = 30 mice). Data shown are mean ± SD. *P* values were calculated by one-way ANOVA (**b**). ** $P<0.01$.
