# Supplementary material for: A novel protein RASON encoded by a lncRNA controls oncogenic RAS signaling in KRAS mutant cancers
Source: Cell Res. 2022 Oct 14;33(1):30–45. doi: 10.1038/s41422-022-00726-7 (PMC9810732; doi:10.1038/s41422-022-00726-7)
Supplement: Supplementary file 10 — Fig. S10 [file 41422_2022_726_MOESM10_ESM.pdf]

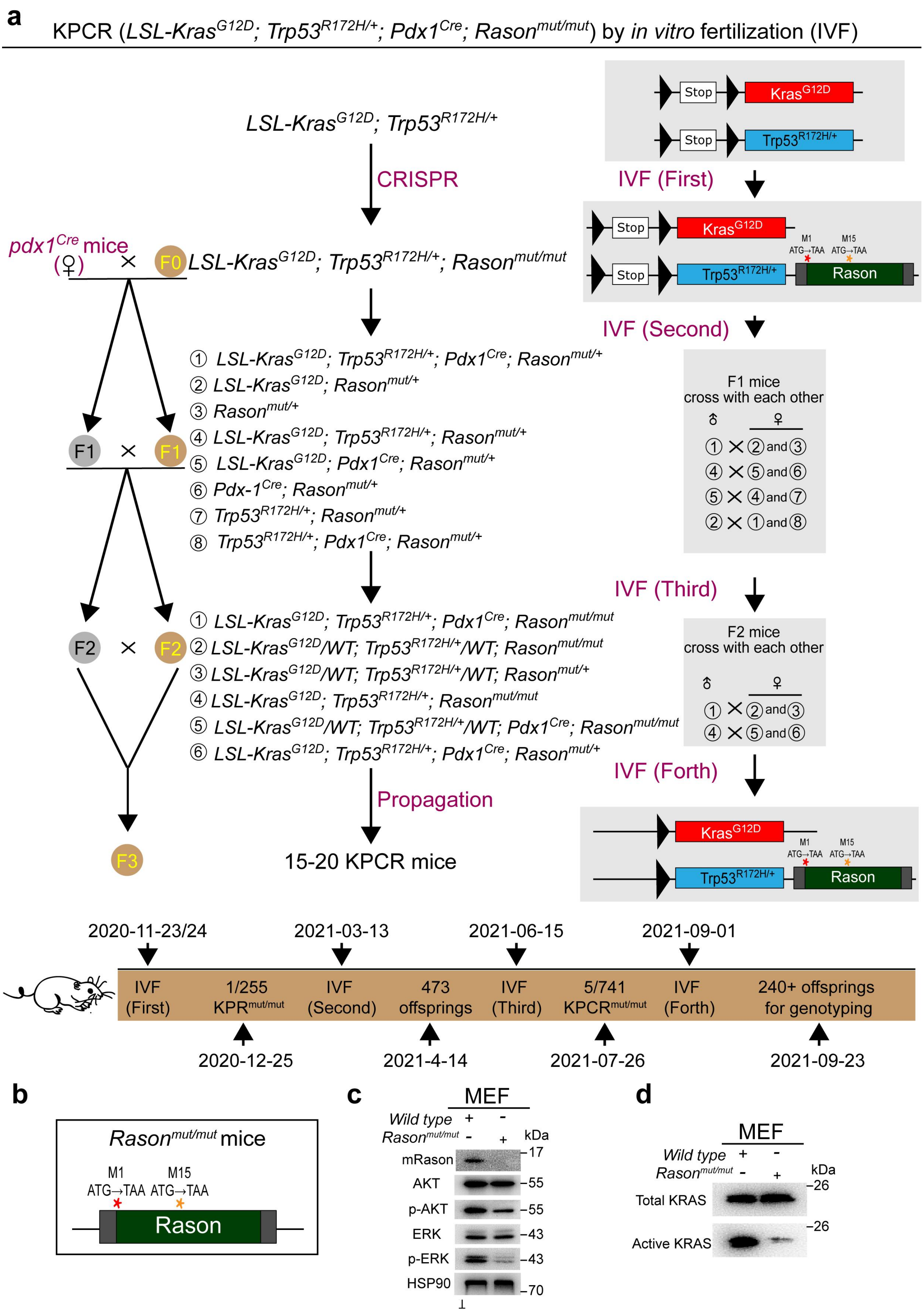
a
KPCR (LSL-Kras G12D; Trp53 R172H/+; Pdx1 Cre; Rason mut/mut) by in vitro fertilization (IVF)
LSL-Kras G12D; Trp53 R172H/+
CRISPR
pdx1 Cre mice (♀)
×
F0
LSL-Kras G12D; Trp53 R172H/+; Rason mut/mut
① LSL-Kras G12D; Trp53 R172H/+; Pdx1 Cre; Rason mut/+
② LSL-Kras G12D; Rason mut/+
③ Rason mut/+
④ LSL-Kras G12D; Trp53 R172H/+; Rason mut/+
⑤ LSL-Kras G12D; Pdx1 Cre; Rason mut/+
⑥ Pdx-1 Cre; Rason mut/+
⑦ Trp53 R172H/+; Rason mut/+
⑧ Trp53 R172H/+; Pdx1 Cre; Rason mut/+
F1
F1
① LSL-Kras G12D; Trp53 R172H/+; Pdx1 Cre; Rason mut/mut
② LSL-Kras G12D/WT; Trp53 R172H/+/WT; Rason mut/mut
③ LSL-Kras G12D/WT; Trp53 R172H/+/WT; Rason mut/+
④ LSL-Kras G12D; Trp53 R172H/+; Rason mut/mut
⑤ LSL-Kras G12D/WT; Trp53 R172H/+/WT; Pdx1 Cre; Rason mut/mut
⑥ LSL-Kras G12D; Trp53 R172H/+; Pdx1 Cre; Rason mut/+
F2
F2
Propagation
F3
15-20 KPCR mice
Stop
Kras G12D
Stop
Trp53 R172H/+
IVF (First)
M1 ATG→TAA
M15 ATG→TAA
Rason
IVF (Second)
F1 mice
cross with each other
♂ ♀
① × ② and ③
④ × ⑤ and ⑥
⑤ × ④ and ⑦
② × ① and ⑧
IVF (Third)
F2 mice
cross with each other
♂ ♀
① × ② and ③
④ × ⑤ and ⑥
IVF (Forth)
2020-11-23/24
2021-03-13
2021-06-15
2021-09-01
IVF (First)
1/255 KPR mut/mut
IVF (Second)
473 offsprings
IVF (Third)
5/741 KPCR mut/mut
IVF (Forth)
240+ offsprings for genotyping
2020-12-25
2021-4-14
2021-07-26
2021-09-23
b
Rason mut/mut mice
M1 ATG→TAA
M15 ATG→TAA
Rason
c
MEF
Wild type + -
Rason mut/mut - +
kDa
mRason -17
AKT -55
p-AKT -55
ERK -43
p-ERK -43
HSP90 -70
d
MEF
Wild type + -
Rason mut/mut - +
kDa
Total KRAS -26
Active KRAS -26

**Supplementary information, Fig. S10 Establishment of the *LSL-Kras*$^{G12D}$; *Trp53*$^{R172H/+}$*; Pdx1*$^{Cre}$; *Rason*$^{mut/mut}$ mouse strain (KPCR mice). a** schematic of KPCR mice generation by *in vitro* fertilization (IVF). *Rason* knockout was introduced into the *LSL-Kras*$^{G12D}$; *Trp53*$^{R172H/+}$*; Pdx1*$^{Cre}$ (KPC) genetic background by mutations of two start codons in the *Rason* sORF (M1X and M15X). **b-d** characterization of *Rason*$^{mut/mut}$ mice. **b** *Rason*$^{mut/mut}$ mice was a byproduct of KPCR mice generation in A. **c** IB showing the status of KRAS effector signaling in MEFs from wildtype and *Rason*$^{mut/mut}$ mice. **d** active KRAS-GTP levels as determined by GST-Raf1-RBD pull-down assay in MEFs from wildtype and *Rason*$^{mut/mut}$ mice.
