# Supplementary material for: A novel protein RASON encoded by a lncRNA controls oncogenic RAS signaling in KRAS mutant cancers
Source: Cell Res. 2022 Oct 14;33(1):30–45. doi: 10.1038/s41422-022-00726-7 (PMC9810732; doi:10.1038/s41422-022-00726-7)
Supplement: Supplementary file 11 — Fig. S11 [file 41422_2022_726_MOESM11_ESM.pdf]

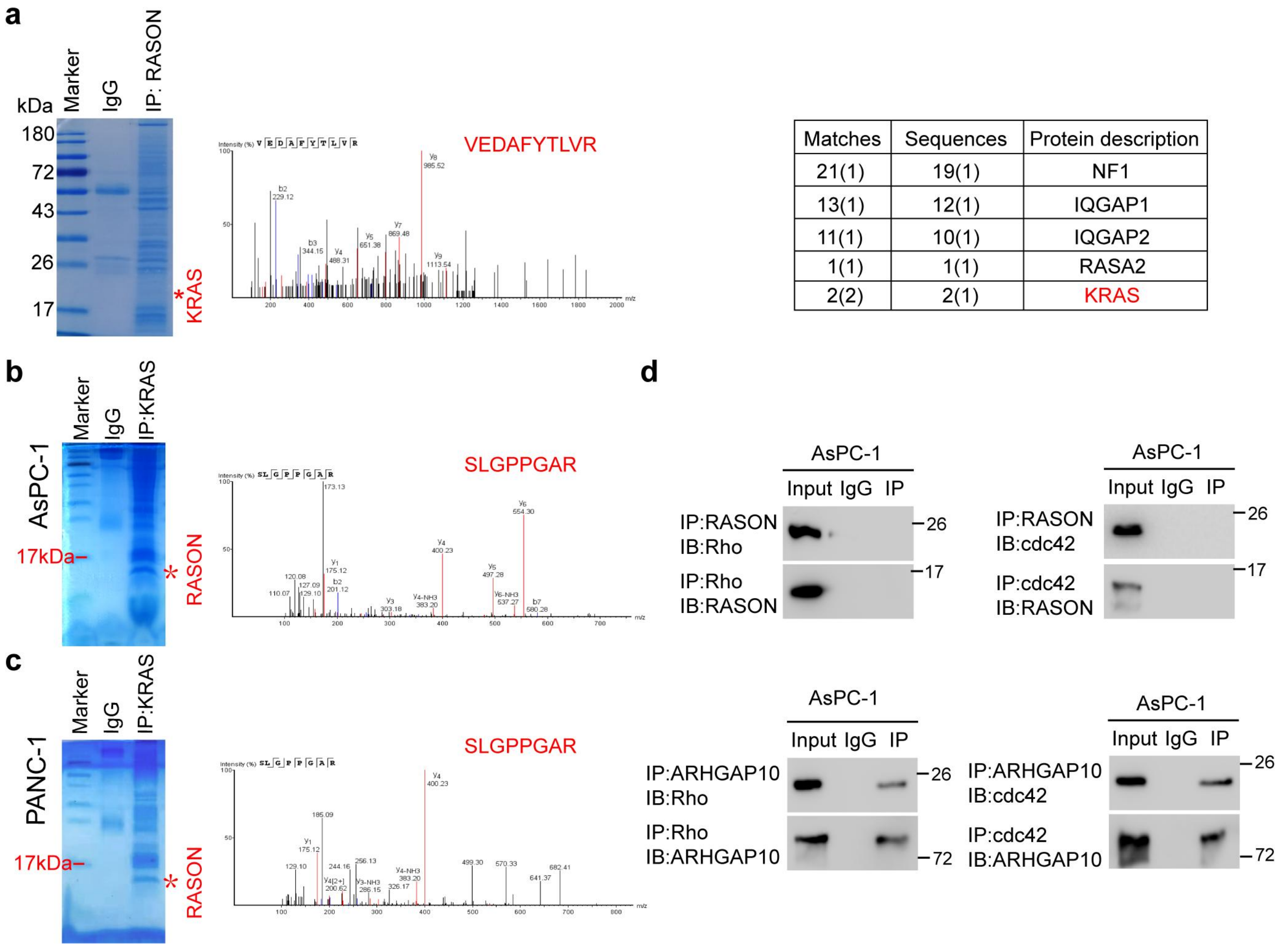

Supplementary information, Fig. S11 RASON binds to KRAS but not Rho or CDC42. a IP-MS detection of RASON binding partners in AsPC-1 cells. **b, c** RASON-specific peptides were detected in IP-MS experiments using KRAS antibody in AsPC-1 (**b**) and PANC-1 (**c**) cells. **d** IP between RASON and two other RAS superfamily members Rho and CDC42 in AsPC-1 cells. ARHGAP10, a GAP protein known to bind to Rho and cdc42, was used as a positive control.
