# Supplementary material for: A novel protein RASON encoded by a lncRNA controls oncogenic RAS signaling in KRAS mutant cancers
Source: Cell Res. 2022 Oct 14;33(1):30–45. doi: 10.1038/s41422-022-00726-7 (PMC9810732; doi:10.1038/s41422-022-00726-7)
Supplement: Supplementary file 12 — Fig. S12 [file 41422_2022_726_MOESM12_ESM.pdf]

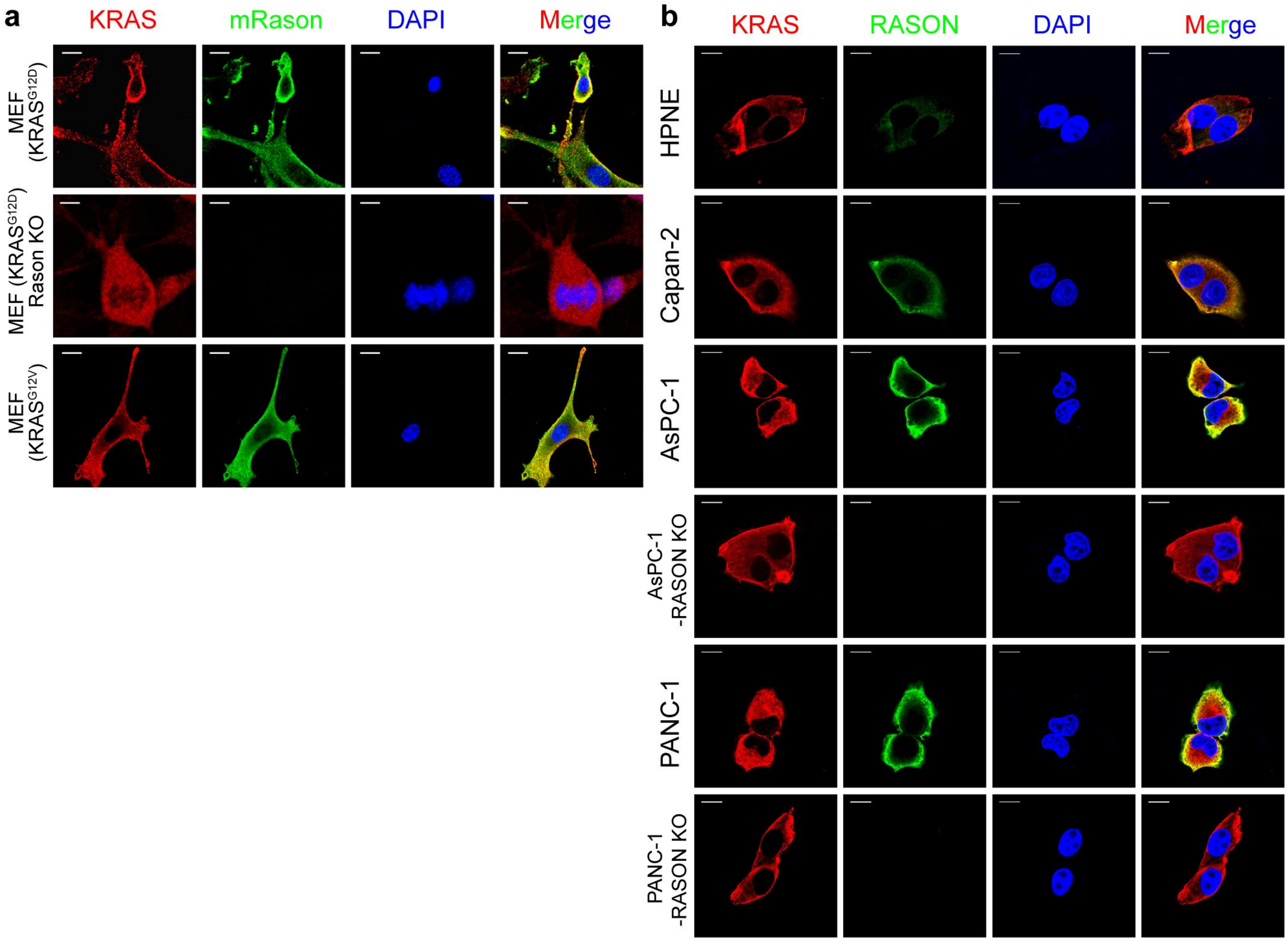

Supplementary information, Fig. S12 Co-localization of RASON and KRAS *in vitro*. a immunofluoresence images showing the co-localization of RASON and KRAS in RAS-less MEFs stably overexpressing KRAS^{G12D} or KRAS^{G12V}. **b** immunofluoresence images showing the co-localization of RASON and KRAS in HPNE normal pancreas and human PDAC cells. Bars, 10 µm.
