# Supplementary material for: A novel protein RASON encoded by a lncRNA controls oncogenic RAS signaling in KRAS mutant cancers
Source: Cell Res. 2022 Oct 14;33(1):30–45. doi: 10.1038/s41422-022-00726-7 (PMC9810732; doi:10.1038/s41422-022-00726-7)
Supplement: Supplementary file 13 — Fig. S13 [file 41422_2022_726_MOESM13_ESM.pdf]

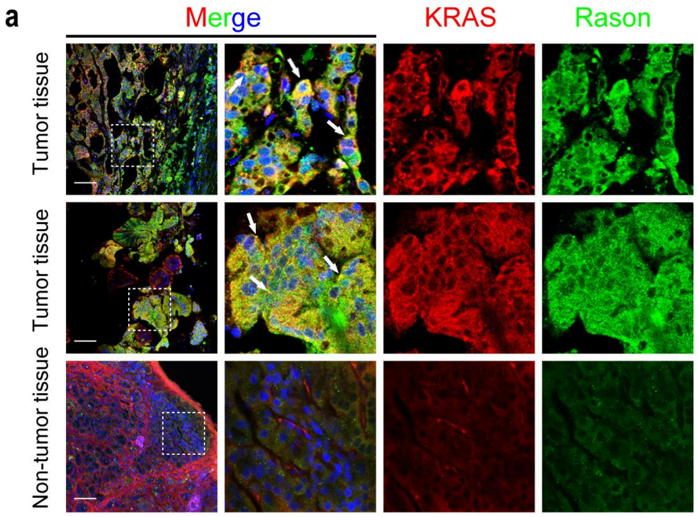

Supplementary information, Fig. S13 Co-localization of RASON and KRAS *in vivo*. a immunofluoresence images showing the co-localization of RASON and KRAS in tumor and normal tissues from PDAC patients. Bars, 50 μm.
