# Supplementary material for: A novel protein RASON encoded by a lncRNA controls oncogenic RAS signaling in KRAS mutant cancers
Source: Cell Res. 2022 Oct 14;33(1):30–45. doi: 10.1038/s41422-022-00726-7 (PMC9810732; doi:10.1038/s41422-022-00726-7)
Supplement: Supplementary file 14 — Fig. S14 [file 41422_2022_726_MOESM14_ESM.pdf]

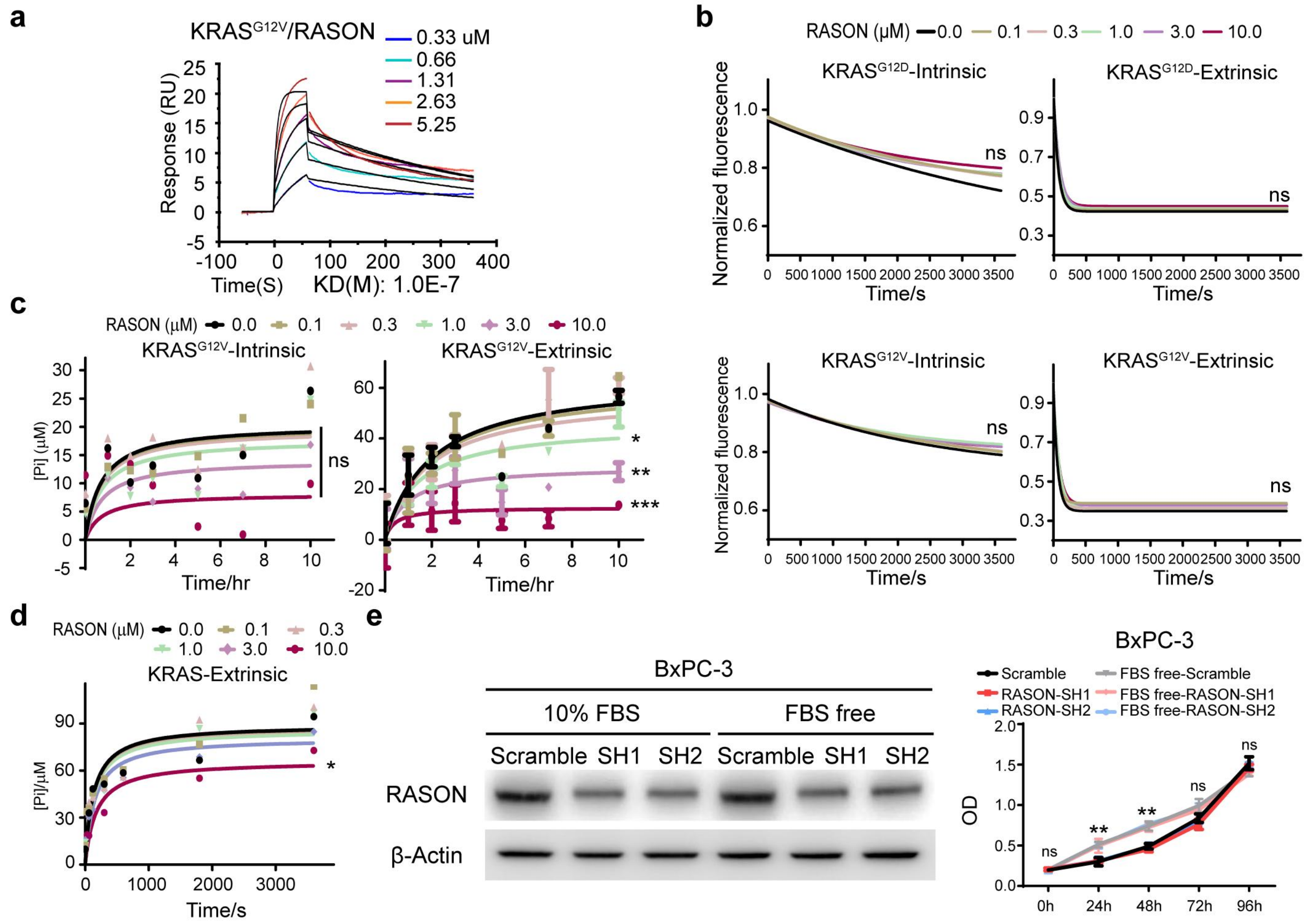

Supplementary information, Fig. S14 RASON binds to KRAS and inhibits GTP hydrolysis. a Surface Plasma Resonance (SPR) analysis of the interaction between RASON and $KRAS^{G12V}$. **b** intrinsic (left) and SOS-catalysed (right) nucleotide exchange of $KRAS^{G12D}$ and $KRAS^{G12V}$ in the presence of RASON (RASON was titrated in 3-fold dilution series). **c** intrinsic (left) and NF1-stimulated (right) $KRAS^{G12V}$ GTPase activity in the presence of RASON were measured using Malachite Green assay (RASON was titrated in 3-fold dilution series). **d** the effect of RASON on extrinsic GTP hydrolysis of wildtype KRAS. **e** the effect of RASON knockdown on the proliferation of BxPC-3 ($KRAS^{WT}$) cells. (left) RASON protein expression levels in BxPC-3 cells with or without RASON knockdown cultured with 10% FBS or serum-free medium for 48 hours. (right) the effect of RASON knockdown on the proliferation of BxPC-3 cells. Cells were cultured with 10% FBS or serum-free medium for 48 hours followed by CCK8 assay in 10% FBS medium. *P* values were calculated by two-way ANOVA (**b**, **c**, **d**, **e**). * $P<0.05$, ** $P<0.01$, *** $P<0.001$.
