# Supplementary material for: A novel protein RASON encoded by a lncRNA controls oncogenic RAS signaling in KRAS mutant cancers
Source: Cell Res. 2022 Oct 14;33(1):30–45. doi: 10.1038/s41422-022-00726-7 (PMC9810732; doi:10.1038/s41422-022-00726-7)
Supplement: Supplementary file 15 — Fig. S15 [file 41422_2022_726_MOESM15_ESM.pdf]

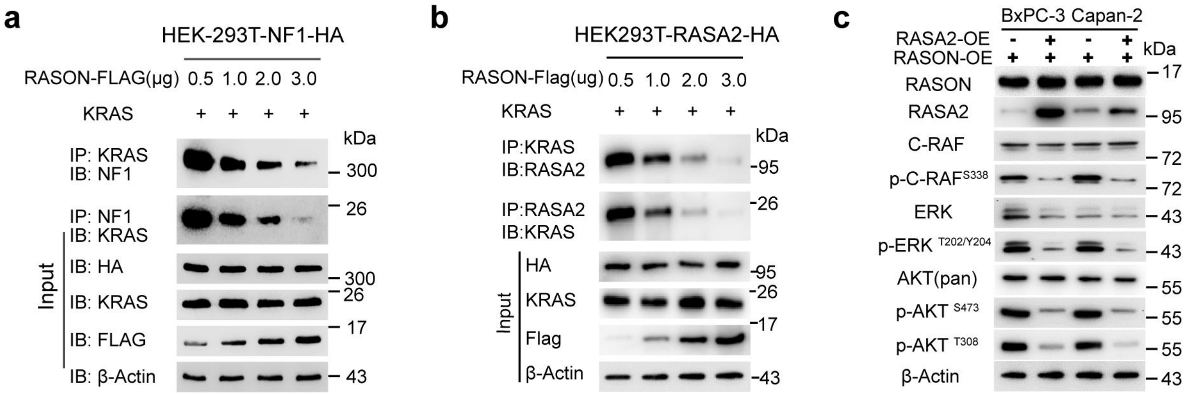

Supplementary information, Fig. S15 RASON could compete with RAS-GAPs. a effect of RASON OE on KRAS-NF1 interaction. HEK293T cells were transiently transfected with increasing levels of FLAG-RASON and same level of KRAS-WT, KRAS-NF1 binding was analyzed by IP. **b** effect of RASON OE on KRAS interaction with RASA2. HEK293T cells were transiently transfected with increasing levels of FLAG-RASON and same level of KRAS-WT, KRAS/RAS-GAP binding was analyzed by IP. **c** IB analysis of KRAS signaling in BxPC-3 and Capan-2 RASON-OE cells with forced RASA2 overexpression.
