# Supplementary material for: A novel protein RASON encoded by a lncRNA controls oncogenic RAS signaling in KRAS mutant cancers
Source: Cell Res. 2022 Oct 14;33(1):30–45. doi: 10.1038/s41422-022-00726-7 (PMC9810732; doi:10.1038/s41422-022-00726-7)
Supplement: Supplementary file 16 — Fig. S16 [file 41422_2022_726_MOESM16_ESM.pdf]

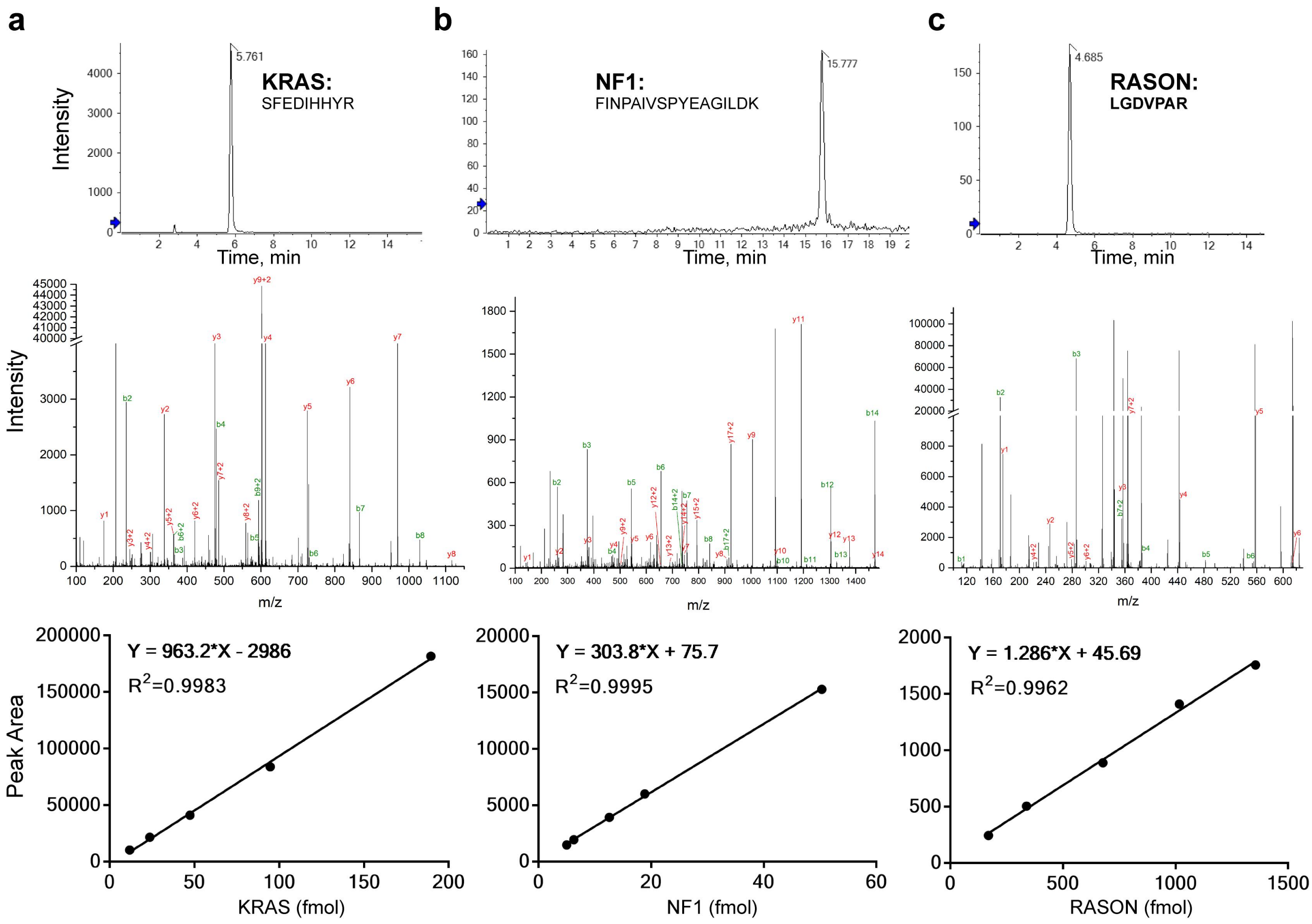

Supplementary information, Fig. S16 Quantification of the concertation of KRAS, NF1 and RASON protein in human PDAC cells. LC-MS methods were used to quantify the protein levels of KRAS, NF1 and RASON in AsPC-1 and PANC-1 pancreatic cancer cell lines. Purified KRAS (**a**), NF1 (**b**) and RASON (**c**) proteins were first used to develop methods and generate a standard curve for each protein. Intracellular levels of each protein were then determined by detection and quantification of the respective signature peptides (top and middle) according to the standard curve (bottom) of each protein.
