# Supplementary material for: A novel protein RASON encoded by a lncRNA controls oncogenic RAS signaling in KRAS mutant cancers
Source: Cell Res. 2022 Oct 14;33(1):30–45. doi: 10.1038/s41422-022-00726-7 (PMC9810732; doi:10.1038/s41422-022-00726-7)
Supplement: Supplementary file 17 — Fig. S17 [file 41422_2022_726_MOESM17_ESM.pdf]

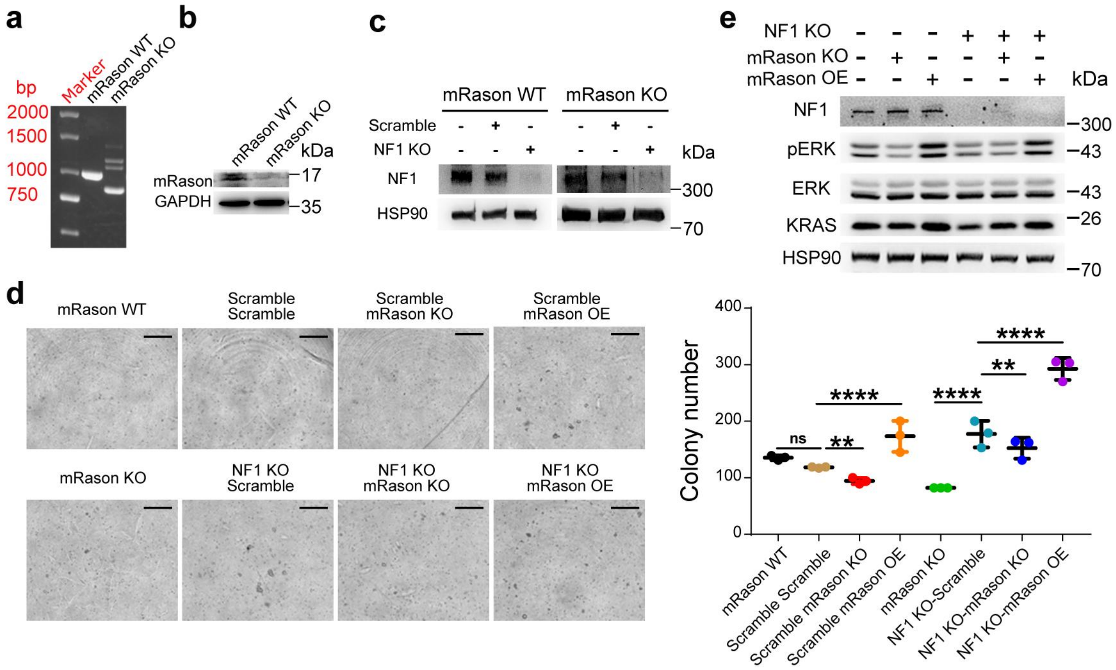

Supplementary information, Fig. S17 Effect of *Nf1* KO plus *Rason* KO/OE on the malignant transformation of $KRAS^{G12D}$ MEF cells. a-c, Manipulation of NF1 and RASON expression in $KRAS^{G12D}$ MEF cells**.** We first knocked out *Nf1* using CRISPR-Cas9 in both MEF^{G12D} and MEF^{G12D}-*Rason*-KO cells; we then overexpressed *Rason* in both MEF^{G12D} and MEF^{G12D}-*NF1*-KO cells. **a** confirmation of *Rason* KO by PCR. **b** confirmation of *Rason* KO by IB. **c** confirmation of *Nf1* KO in MEF cells with or without *Rason* KO. **d** A total of eight cell lines – $KRAS^{G12D}$ MEF; MEF (Scramble; Scramble); MEF (Scramble; *Rason* KO); MEF (Scramble; *Rason* OE); MEF (*Rason* KO); MEF (*Nf1* KO; Scramble); MEF (*Nf1* KO; *Rason* KO); MEF (*Nf1* KO; *Rason* OE) – were compared for their ability to form 3-D colonies *in vitro* (bars, 1 mm). Representative images from each group were shown (left) with quantifications (right). **e** IB showing the status of KRAS effector signaling in sub-cu tumors derived from $KRAS^{G12D}$ MEFs with the indicated genetic manipulations. Data in dot graphs represent mean ± SD. *P* values were calculated by one-way ANOVA (**d**). ** *P*<0.01, **** *P*<0.0001.
