# Supplementary material for: A novel protein RASON encoded by a lncRNA controls oncogenic RAS signaling in KRAS mutant cancers
Source: Cell Res. 2022 Oct 14;33(1):30–45. doi: 10.1038/s41422-022-00726-7 (PMC9810732; doi:10.1038/s41422-022-00726-7)
Supplement: Supplementary file 18 — Fig. S18 [file 41422_2022_726_MOESM18_ESM.pdf]

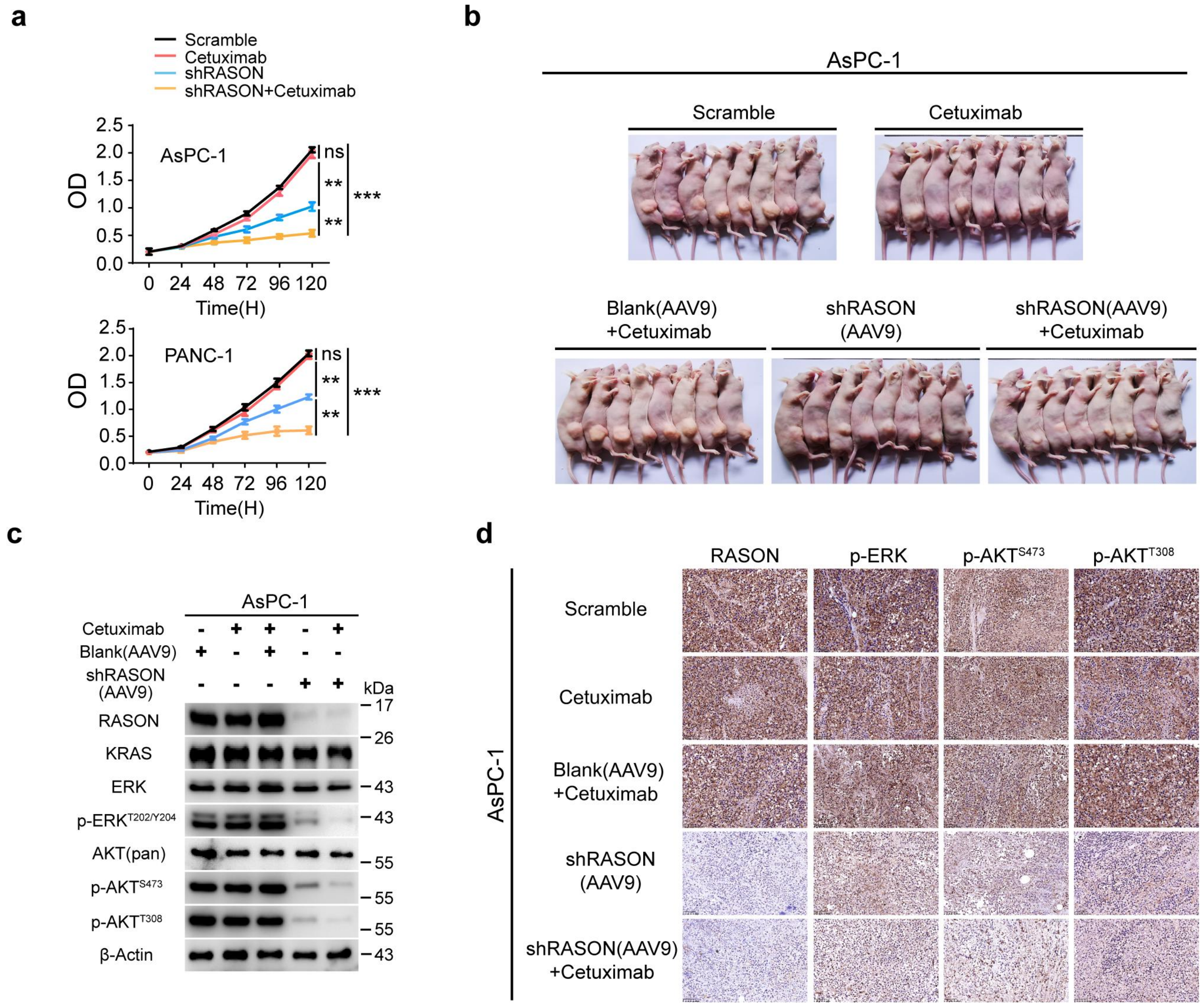

Supplementary information, Fig. S18 RASON knockdown sensitizes AsPC-1 cells to cetuximab. a synergetic effect of RASON KO and cetuximab treatment on the proliferation of AsPC-1 and PANC-1 cell lines *in vitro*. Cells were treated with cetuximab, RASON shRNA, or both, cell growth was measured every 24 h for 5 days. **b** images of AsPC-1 xenograft tumors following treatement with scramble, cetuximab, shRASON, or both. **c, d** immunoblots and immunohistochemistry showing the effects on KRAS effector signaling in AsPC-1 xenograft tumors with indicated treatments. Data in line graphs represent mean ± SD. *P* values were calculated by two-way ANOVA (**a**). ** $P<0.01$, *** $P<0.001$.
